# Interactions of multiple rhythms in a biophysical network of neurons

**DOI:** 10.1186/s13408-020-00096-7

**Published:** 2020-11-17

**Authors:** Alexandros Gelastopoulos, Nancy J. Kopell

**Affiliations:** 1 Department of Mathematics and Statistics, Boston University, 111 Cummington Mall, 02215 Boston, MA USA; 2 Department of Marketing and Management, University of Southern Denmark, Campusvej 55, 5230 Odense, Denmark

**Keywords:** Neural oscillations, Phase-locking, Phase-response curve, State reset, Time-division multiplexing

## Abstract

Neural oscillations, including rhythms in the beta1 band (12–20 Hz), are important in various cognitive functions. Often neural networks receive rhythmic input at frequencies different from their natural frequency, but very little is known about how such input affects the network’s behavior. We use a simplified, yet biophysical, model of a beta1 rhythm that occurs in the parietal cortex, in order to study its response to oscillatory inputs. We demonstrate that a cell has the ability to respond at the same time to two periodic stimuli of unrelated frequencies, firing in phase with one, but with a mean firing rate equal to that of the other. We show that this is a very general phenomenon, independent of the model used. We next show numerically that the behavior of a different cell, which is modeled as a high-dimensional dynamical system, can be described in a surprisingly simple way, owing to a reset that occurs in the state space when the cell fires. The interaction of the two cells leads to novel combinations of properties for neural dynamics, such as mode-locking to an input without phase-locking to it.

## Introduction

Neural oscillations are ubiquitous in the brain and are thought to play important roles in cognition [1–4]. Different brain networks often communicate with one another via neural oscillations [5–7]: periodic firing of the neurons of one network can serve as input to another network. Thus, studying how an oscillatory network responds to periodic input is important for understanding information processing in the brain.

The simplest form of response to a periodic input is simple entrainment, where the network picks up the rhythm of the input [8]. Different types of responses can be described using the phase-response curve (PRC) method [9]. In its simplest version, the PRC describes the time that an oscillating cell fires as a function of the phase when it receives a stimulus. A different approach is to calculate the spike-triggered average [10], that is, the average input in a window of time before a spike occurs. These and similar methods have been widely applied to characterize the response of both model and real neurons.

However, the response of an entire network to an input can be more complex than the responses of its individual cells. Individual cell properties interact with the network topology and even the characteristics of the synapses. Here we take a different approach: we start with a network that produces a surprising response to an oscillatory input and, using a variety of methods and simplifications, we dissect the network to find the essential properties that give rise to the observed behavior.

There is a large variety of brain rhythms and mechanisms that produce them [1]. Here we focus on a rhythm of frequency about 15 Hz (beta1 band) that appears in the parietal cortex [11, 12], because of its unusual and functionally important dynamics, as well as the fact that there exists a biophysical computational model for it. An important property of this rhythm is that the beta1 period arises from a concatenation of two shorter cycles of physiologically relevant lengths: a beta2 ($\sim 40\text{ ms}$) and a gamma ($\sim 25\text{ ms}$) timescale. Moreover, in the absence of any input, the existence of the beta1 rhythm depends on the interactions among all cell types considered in the model. Thus, one would expect that driving a subset of the network at a frequency other than beta1, in particular at one of those faster timescales (gamma or beta2), would destroy the beta1 rhythm in the whole network. Surprisingly, in [13] we observed that in some cases the beta1 rhythm can persist, despite a subset of the network being driven by a 40 Hz oscillatory input. The input oscillation and the natural beta1 rhythm interact in a non-trivial way, with the principal cells that sustain the beta1 rhythm firing always out of phase with the incoming pulses.

In the current work we aimed to characterize the interaction of the two rhythms (gamma and beta1) and explain the mechanism that supports this interaction. Although certain aspects of the behavior did depend on physiological details of the individual cells, there were other aspects that were consequences of a combination of very simple properties of the cells, synapses, and the network topology. Thus, parts of the mechanism are much more general than this particular network and reproducible by simpler neuron models, even by abstract models that make no mention of neurons. The next paragraphs give an overview of our results.

At the crux of the interaction of the input gamma with the natural beta1 rhythm lies a certain cell (SI cell, for Slow Inhibitory), which in the network happens to be stimulated by two roughly periodic sources, and is able to fire in phase with the first, but at a rate equal to the second. After applying successive simplifications to the model and showing that the reduced versions still reproduce the surprising aspects of the SI cell’s behavior, we arrive at an explanation of this behavior based on a few very simple properties. In particular, we show that the combination of a fast pulsatile excitatory input and an independent slower inhibitory input can make a cell fire in phase with the faster input, but at a rate equal to the slower input. Moreover, the cell fires only in a window of phase of the slower input. These properties are robust to deviations of the inputs from being purely periodic. The only required properties of the cell are that it fires only immediately after the excitatory input’s pulses, that it cannot fire on two consecutive pulses, and that it cannot fire for some time when it is inhibited. That these properties are enough is confirmed by construction of a mathematical model with such properties that can reproduce the cell’s behavior. The mechanism is novel in the sense that it combines aspects of both simple entrainment of a cell to an (excitatory) input [8] and of inhibition-based rhythms [14], but differs from both in fundamental ways (see Discussion).

We next show mathematically that the above hold true for certain ranges of frequencies for the two inputs. These ranges are only relative to each other and to other model parameters, meaning that there are no absolute bounds on the frequencies. We confirm that the mathematics accurately describe the behavior of the cell in the network by showing simulation results with varying parameters.

Next we illustrate that another cell (IB cell, for Intrinsically Bursting), which is modeled as a high-dimensional dynamical system, can be approximately described by a PRC-like map [15]. A basic assumption of the PRC method is that the cell is on or sufficiently close to its limit cycle when the stimulus arrives. The IB cell involves several state variables with time constants longer than the interval between successive stimuli it receives, so there is in principle no reason why it should return to its limit cycle in the time between stimuli.^1^ Still, the time that the cell fires is approximately determined by the timing of the last stimulus only, making a PRC-like description possible. Based on numerical results, we show that the reason for this is a reset of its state that occurs when the cell fires, causing it to effectively forget much of its history. Unlike in some simpler models [16], this reset is not hard-coded in our model, but a consequence of the dynamics that occur during an action potential.

In certain parameter regimes, an additional interesting phenomenon takes place: while the cell fires consistently *p* times in every *q* cycles of a periodic input for some integers $p,q$ (a property called *mode-locking* [17]), the phases at which it fires vary significantly (that is, no *phase-locking*), even in the absence of any noise. Our analysis suggests that the reason for this is that the number of spikes in a burst of the IB cell is highly sensitive to the cell’s state at the time it receives a stimulus. A similar sensitivity has been observed in studies of the Hindmarsh–Rose neuronal model [18, 19] and explained through the PRC. In our case PRC theory fails to explain the phenomenon.

Finally, the dynamics of the SI and IB cells together can help explain another property of our network: the two cells consistently fire alternately (for certain parameter ranges), and when the network is excited by a periodic input, their common firing rate is often a rational multiple of the input’s frequency, even in cases when the IB cell is not firing periodically (see previous paragraph).

Regarding the implications of our results for brain function, in [13] we have argued that the ability of this system to process a new rhythm while retaining an established one, combined with other properties, makes it a good candidate for a physiological substrate of a working-memory buffer [20]. The details of the mechanism studied in the current paper suggest time-division multiplexing as another possible function. Multiplexing is a term in telecommunications that refers to the use of the same channel for transmitting signals from multiple sources. In time-division multiplexing, time is divided into intervals during each of which a single source makes exclusive use of the channel. In the application we describe, a single neuron transmits information from different sources at different times. Such a function can be relevant for the parietal cortex, which is known to combine inputs from multiple sources [21].

The structure of the paper is as follows: in Sect. 2 we describe the computational model. In Sect. 3 we show simulation results that highlight the SI cell behavior and demonstrate that this behavior can be reproduced by simpler models. In Sect. 4 we give mathematical results that explain the SI cell behavior and explore some of their predictions. In Sect. 5, using a combination of analytical and numerical methods, we study the dynamics of the IB cell and show how these dynamics, combined with our understanding of the SI cell from before, can explain the interaction of the two cells with each other and with the periodic input. The application to time-division multiplexing is described in the Discussion.

## The model

### Overview and network topology

**Figure 1:**
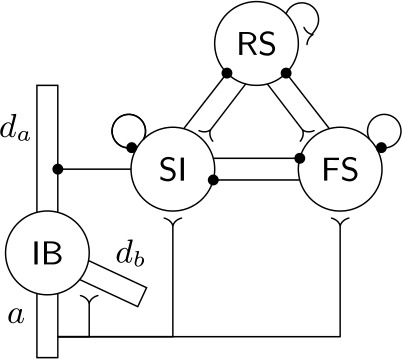
The network model. Large circles denote neurons and lines denote synapses. Filled circles (for inhibitory synapses) or inverted arrows (for excitatory synapses) specify the postsynaptic (target) cell. In the IB cell we distinguish four compartments: the soma (circle in the middle), the axon (*a*), the apical dendrite ($d_{a}$), and the basal dendrite ($d_{b}$)

We use an adapted version of a model for the parietal beta1 rhythm [12]. It consists of a network of Hodgkin–Huxley type neurons, of four different types. Although in the original model there were multiple cells of each type, the results were reproducible when a single cell of each type was considered. Here, to simplify the analysis, we consider the simpler version with a single cell of each type. The connectivity is as shown in (Fig. 1).

The four cells in our model are the following: regular spiking (RS), fast-spiking (FS), slow inhibitory (SI), and intrinsically bursting (IB) neurons. The connectivity is shown in Fig. 1. As in [12], the cells have no geometry, except that the IB cell consists of four compartments: a soma, an axon, an apical dendrite, and a basal dendrite. Each compartment follows Hodgkin–Huxley dynamics and different compartments are connected via electrical coupling. All other cells are single compartments.

We now give a brief description of the cells (see Sect. 2.2 for details). The RS and IB cells are excitatory, while the FS and SI cells are inhibitory. The FS cell is the simplest one, containing no currents except for the standard Hodgkin–Huxley currents (leak current, transient sodium current, and delayed rectifier potassium current). It is fast to spike when excited and its (outgoing) synapses have relatively fast rise and decay times. The SI cell in turn is slower to spike when excited, and its synapses have slower decay times. Apart from the standard currents, it also contains an h-current, which is a depolarizing current that slowly builds up when the cell is not firing (and builds up faster if the cell is hyperpolarized), but quickly returns to small values when the cell fires. This results in the SI cell being less excitable than normal for tens of milliseconds after it fires. The RS is a regular excitatory cell with the addition of an h-current as well. The various IB compartments contain several non-standard currents (see Sect. 2.2), the combination of which gives the IB cell rich dynamics, including making it a bursting cell.

Below we give the equations that describe the dynamics of each cell/compartment, the synapses, and the inputs. We note, however, that much of the behavior of the network does not depend on these details, as is evident in Sects. 3 and 4. Details of the cell dynamics, in particular those of the IB cell, will become relevant in Sect. 5. The model and parameters are taken from [12] (with minimal modifications, see Appendix D.1). The reason that we start with this model, instead of a simpler one that can reproduce much of the behavior, is to show that it can exhibit this simple behavior *despite* its complexity.

### Equations

We start with the general form of the equations for each cell/compartment. The membrane potential **V** follows the equation (we use boldface characters for state variables)

1 $$\begin{aligned} C\cdot \frac{d\mathbf{V}}{dt}={}& {-}J-I_{\mathrm{syn}}-I_{el}-I_{\mathrm{ext}} \\ &{} -g_{L}\cdot (\mathbf{V}-V_{L}) \\ &{} -g_{Na}\cdot m_{0}^{3}(\mathbf{V})\cdot \mathbf{h}\cdot (\mathbf{V}-V_{Na}) \\ & {}-g_{K}\cdot \mathbf{m}^{4}\cdot (\mathbf{V}-V_{K}) \\ &{} -g_{AR}\cdot \mathbf{m}_{AR}\cdot (\mathbf{V}-V_{AR}) \\ &{} -g_{KM}\cdot \mathbf{m}_{KM}\cdot (\mathbf{V}-V_{KM}) \\ &{} -g_{CaH}\cdot \mathbf{m}_{{CaH}}^{2}\cdot ( \mathbf{V}-V_{CaH}). \end{aligned}$$

Here *C*, *J*, $g_{x}$, and $V_{x}$, where $x=L,Na,K,AR,KM,CaH$, are all constants, and $m_{0}(\mathbf{V})$ is a function of **V**. The term *J* models background excitation, $I_{\mathrm{syn}}$ and $I_{el}$ depend on the membrane potentials of other cells/compartments and are described below (Eqs. (3) and (4)), while $I_{\mathrm{ext}}$ is an externally controlled input.

Each line after the first line models one ionic current. The first three are the standard currents of a Hodgkin–Huxley model: L - leak current, Na - transient sodium current, K - delayed rectifier potassium current. The rest of the currents, present only in certain cells, are as follows: AR is for anomalous rectifier current, also called h-current, KM for M-current, CaH for high-threshold calcium current. Not all non-standard currents are present in all of the cells/compartments. The h-current is present in the RS and SI cells, and the IB dendritic compartments; the M-current is present in the IB axon and dendritic compartments; and the high-threshold calcium current is present only in the IB dendrites.

The gating variables follow first order dynamics, but with their equilibria and time constants depending on **V**. More precisely, we have

2 $$\begin{aligned} \frac{d\mathbf{x}}{dt}=\frac{1}{\tau _{x}(\mathbf{V})}\cdot \bigl( x_{ \infty }( \mathbf{V})-\mathbf{x} \bigr), \quad\text{where } \mathbf{x}=\mathbf{m}, \mathbf{h}, \mathbf{m}_{AR},\mathbf{m}_{KM},\mathbf{m}_{CaH}, \end{aligned}$$

and $\tau _{x}(\mathbf{V})$ and $x_{\infty }(\mathbf{V})$ are functions of the membrane potential **V**.

The term $I_{el}$ in Eq. (1) models direct electrical coupling between different compartments and is a sum of terms of the form (one for each compartment that the compartment in question is coupled to)

3 $$ g\cdot \bigl(\mathbf{V}-\mathbf{V}' \bigr), $$

where *g* is a constant and $\mathbf{V}'$ is the membrane potential of the other compartment involved in this electrical coupling.

The term $I_{\mathrm{syn}}$ in Eq. (1) models chemical coupling (chemical synapses) between cells/compartments and is a sum of terms of the form

4 $$ \mathbf{s}\cdot g\cdot (\mathbf{V}-V_{0}), $$

one for each incoming synapse, where *g* and $V_{0}$ are constants and **s** is the synaptic state variable associated with this synapse. The synaptic state variables follow first order dynamics that depend on the presynaptic membrane potential. More specifically,

5 $$ \frac{d\mathbf{s}}{dt}=-\frac{\mathbf{s}}{\tau _{d}}+ \frac{1-\mathbf{s}}{\tau _{r}} \biggl(1+ \tanh \frac{\mathbf{V}_{\mathrm{pre}}}{10} \biggr), $$

where $\tau _{d}$ and $\tau _{r}$ are constants, $\mathbf{V}_{\mathrm{pre}}$ is the membrane potential of the presynaptic cell (measured in mV), and $\tanh (\cdot )$ denotes the hyperbolic tangent function.

The term $I_{\mathrm{ext}}$ in Eq. (1) models an externally applied current. Similarly to $I_{\mathrm{syn}}$, $I_{\mathrm{ext}}$ will also be a sum of currents of the form of Eq. (4), but the dynamics of the state variable **s** will depend on an external potential $\mathbf{V}_{\mathrm{ext}}$, instead of the membrane potential of a presynaptic cell. That is,

6 $$ \frac{d\mathbf{s}}{dt}=-\frac{\mathbf{s}}{\tau _{d}}+ \frac{1-\mathbf{s}}{\tau _{r}} \biggl(1+ \tanh \frac{\mathbf{V}_{\mathrm{ext}}}{10} \biggr). $$

In all cases $\mathbf{V}_{\mathrm{ext}}$ will be pulsatile and approximately periodic. More precisely, its dynamics are described by

7 $$ \frac{d\mathbf{V}_{\mathrm{ext}}}{dt}=-70-\mathbf{V}_{\mathrm{ext}}+ ( 25- \mathbf{V}_{\mathrm{ext}} ) \cdot \sum_{i}\delta (t-t_{i}), $$

where $t_{i}$ are the times of the pulses, $\delta (\cdot )$ denotes the Dirac delta function, $V_{\mathrm{ext}}$ is measured in mV and time in ms. The interpulse intervals $t_{i+1}-t_{i}$ are normally distributed, independently for different *i*’s, with mean $\frac{1}{f}$ and standard deviation $\frac{\sigma }{f}$, where *f* is the nominal frequency and $\sigma \geq 0$ some constant ($\sigma =0$ corresponding to an exactly periodic input).

The values for all constants and the functions $m_{0}(\mathbf{V})$, $\tau _{x}(\mathbf{V})$, $x_{\infty }(\mathbf{V})$, $\alpha _{x}(\mathbf{V})$, and $\beta _{x}(\mathbf{V})$ are given in Appendix D.1.

## Simulation results and reducing the model

### Beta1 oscillation

**Figure 2:**
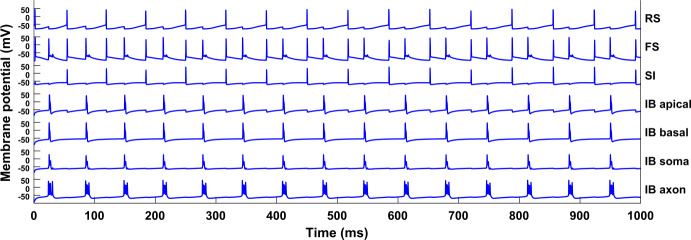
Simulation of the network shown in Fig. 1. Each of the seven blue traces shows the membrane potential of one cell/compartment. All cells fire periodically, with the RS, SI, and IB cells having a frequency of about 15 Hz, and the SI and RS cells being in phase with each other. Parameter values used for all simulations are given in Appendix D.1

Figure 2 shows the membrane potentials of the cells for a simulation of the network without any input. A clear periodicity can be seen, with the RS, SI, and IB cells firing at 15 Hz, but with the IB cell out of phase from the other two, and the FS cell firing at double the rate, in phase with both the IB cell and the RS-SI pair. Also note that when the IB cell fires, its axon bursts, i.e. it fires several spikes in a row.

A version of this network with multiple cells of each type is studied in [12] and it is shown that the pattern of activity is as follows: 1) the RS cell fires and excites the FS and SI cells, 2) the SI cell inhibits the IB cell, which later fires by rebound from inhibition, 3) the IB excites the FS cell, 4) the FS cell inhibits the RS cell which later fires by rebound from inhibition. It follows that the beta1 rhythm, in the absence of any input, relies on the interaction and the phase relations between all types of cells involved in the model.

### Response to oscillatory input

**Figure 3:**
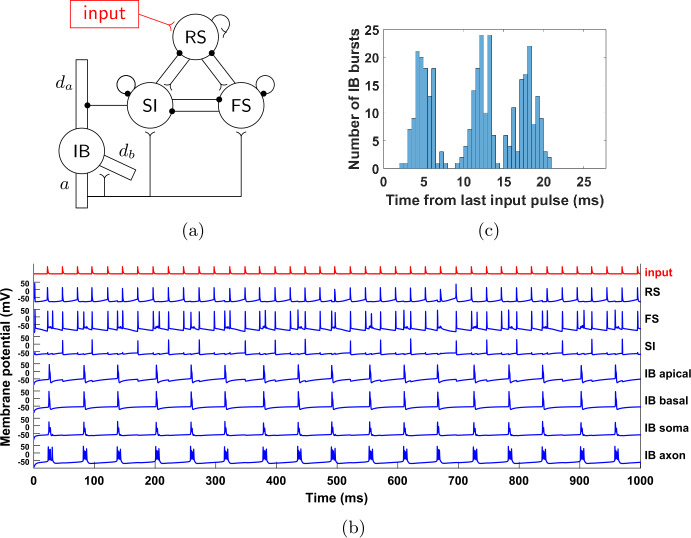
(**a**) A pulsatile 40 Hz input is given to the RS cell. (**b**) Same as Fig. 2, but with the RS cell driven by the input (red trace). The SI cell still fires in phase with the RS cell, but its firing rate remains in the beta1 range (∼17 Hz). (**c**) Histogram of input phase (time from last pulse) when IB cell fires. For each bin, the height of the bar is the number of IB bursts in the simulation having the property that the time elapsed from the last input pulse to the first spike of the burst falls in that bin. The IB cell tends to fire more in certain phases of the external input, showing that the latter is having an effect on the former. Here and in all statistical analysis below, transients are ignored (see Appendix D.2)

Since the beta1 rhythm relies on the interactions among all cells, we might expect that if one of the cells was driven by an external input at a frequency other than beta1, that would destroy the beta1 rhythm from the whole network. However, as shown in Fig. 3(b), this is not the case. Here, we give an excitatory input of frequency 40 Hz (a gamma input) to the RS cell. While the RS cell fires in synchrony with the gamma input that is driving it, the SI and IB cells continue to fire at a rate of about 15 Hz. This is similar to what was seen in the larger network of [13].

Can it be the case that the input does not “reach” the SI and IB cells, so that in the presence of the input they continue what they were doing without it? A closer look at Fig. 3(b) shows that, at least for the SI cell, this is not the case, since it always fires in phase with the input and in fact it fires three times per seven input cycles. The IB cell, on the other hand, fires always out of phase, but its bursts are widely distributed with respect to the input phase (Fig. 3(c)). Moreover, the number of input pulses between successive IB bursts is not constant (Fig. 3(b)), although the cell avoids firing near those pulses. Finally, note that the IB and SI cells fire alternately, which means that the IB cell also fires at 3/7 of the input frequency. All these observations suggest that there is a complex interaction between the SI and IB cells and the input. Our goal is to understand this interaction. It will turn out that both the SI and IB cell have surprising dynamical features.

### The FS-SI-IB network

**Figure 4:**
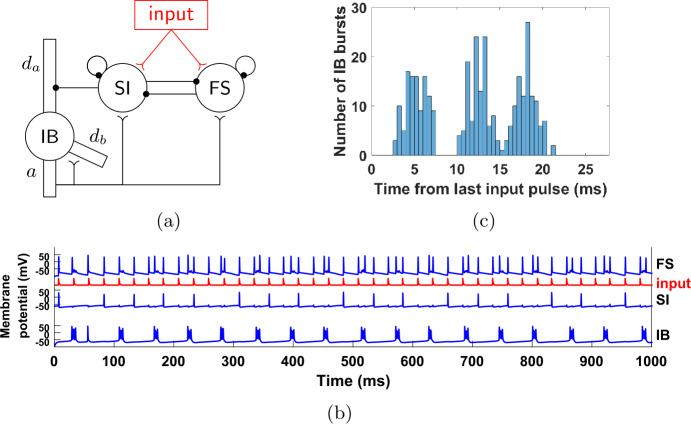
The reduced, 3-cell network. (**a**) Compared to the network of Fig. 3(a), we have removed the RS cell and applied the 40 Hz input directly to the FS and SI cells, via synapses with the same dynamics as the RS→FS and RS→SI synapses, respectively (see Sect. 2.2, Table 5). (**b**) Membrane potential of the three cells (for the IB cell, the membrane potential of the axonal compartment is shown). The behavior is qualitatively the same as in Fig. 3(b). (**c**) Histogram of input phase when the IB cell fires. The results are similar to Fig. 3(c)

To simplify the analysis of our model, we get rid of some complexity that does not contribute to the dynamics. For example, as seen in Fig. 3(b), the RS cell is acting as a relay of the gamma input, with the RS cell firing immediately after receiving a gamma input pulse. Thus, we may remove the cell and connect the gamma input directly to the RS cell’s targets. This is illustrated in Fig. 4(a). Figures 4(b) and (c) show that the dynamics of this simplified network are the same as the initial network; in particular both the SI and IB cells fire three times per seven input cycles, they fire alternately, with the SI firing in phase with the input, while the IB cell fires out of phase but in a wide range of phases.

We will return to this network in Sect. 5, but we first consider a further simplification.

### Further simplifications

#### The FS-SI network with two inputs

Recall from Fig. 3(b) (also evident in Fig. 4(b)) that, although the SI cell fires in phase with the gamma input and the IB cell does not, they have the same firing rate and in fact always fire alternately. It is then natural to ask whether this is a coincidence or if there is a mechanism that makes the two cells have equal firing rates.

To answer this question, we would like to control one of the cells externally and look at the response of the other. The IB cell exhibits the simpler behavior, despite the fact that it is described by a more complicated model (four compartments instead of one). Indeed, the IB cell fires approximately periodically, at a beta1 rate (Fig. 4(b)). Therefore, as a first approximation, we substitute this cell by an oscillatory input of the same frequency. We then vary the frequency of this input, to explore its effect on the other cells, in particular on the SI cell.

**Figure 5:**
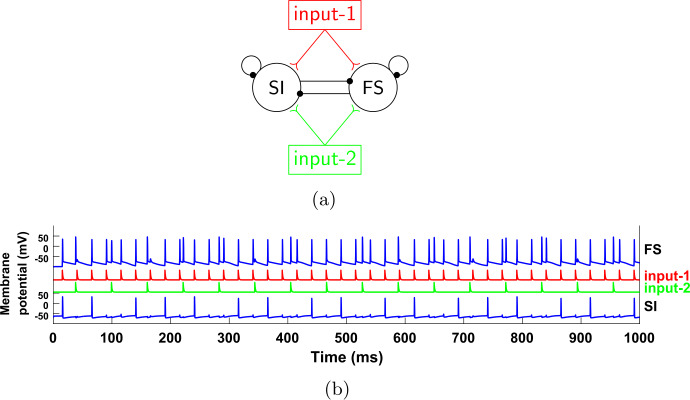
The reduced, 2-cell network. (**a**) Compared to the network in Fig. 4(a), we have replaced the IB cell by an external oscillatory input of frequency 16.357 Hz (frequency chosen so that the ratio of the two input frequencies is not a rational number with a small denominator). This input consists of a single short pulse at the beginning of the period (see Sect. 2.2 and Appendix D.1); we do not model the bursting behavior of the IB cell. The effect of the input onto the FS and SI cells is mediated through excitatory synapses with the same dynamics as the IB→FS and IB→SI synapses before (see Sect. 2.2 and Appendix D.1). (**b**) Membrane potentials of the cells for a simulation of the network shown in (**a**)

Figure 5(a) shows the new reduced network, with the IB cell removed, together with any connections to and from this cell; in its place we have added a beta1 oscillator, which provides input to the other two cells. Note that now we have two sources of periodic input, both given to both the FS and SI cells. The first (input-1) has a gamma frequency and is the same as the one in Fig. 4, while the second (input-2) has a beta1 frequency and plays the role of the IB cell of the network in Fig. 4. Figure 5(b) shows a simulation of this reduced network. Notice that, similarly to Fig. 4, the SI cell fires in phase with input-1 and at a rate equal to that of input-2.

**Figure 6:**
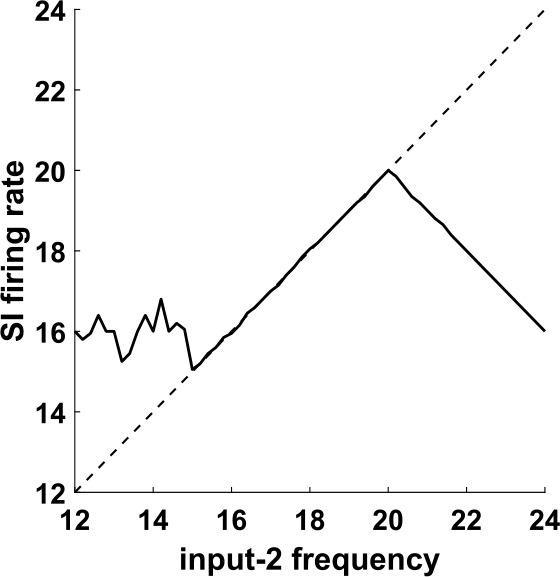
Firing rate of the SI cell as a function of the input-2 frequency, for the network of Fig. 5. For a range of frequencies, the firing rate of the SI cell is equal to the input-2 frequency. Dashed line: diagonal

**Figure 7:**
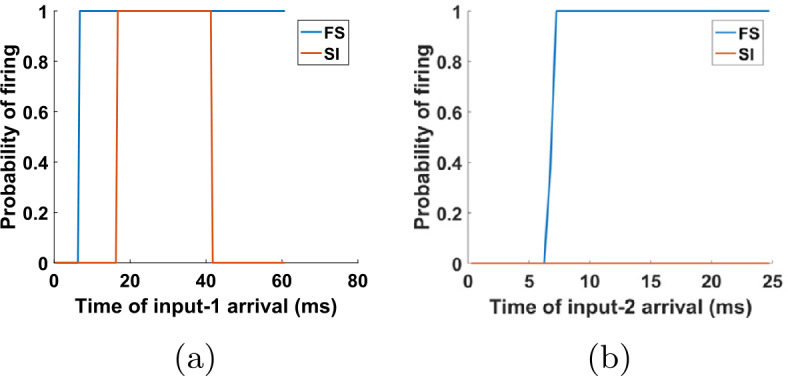
Input phases at which the cells of the network of Fig. 5 fire. (**a**) Probability of firing for FS and SI cells immediately after an input-1 pulse, as a function of when in the input-2 period the input-1 pulse arrives. The horizontal axis measures the time from the last input-2 pulse. The FS cell always fires immediately after an input-1 pulse, unless this pulse occurs within about 5 ms after an input-2 pulse. The SI cell fires immediately after an input-1 pulse, if and only if the last input-2 pulse was between approximately 16–41 ms. (**b**) Same as (**a**), but with the roles of input-1 and input-2 reversed. The FS cell fires at every input-2 pulse, unless it arrives within 5 ms after an input-1 pulse. In contrast, the SI cell never fires immediately after an input-2 pulse

Recall that we have substituted the IB cell by input-2, in order to be able to control its frequency externally and check whether the equality between this frequency and the frequency of the SI will continue to hold. Figure 6 shows that this is the case for a range of input-2 frequencies. This is very surprising, because the SI cell, when it fires, it fires in response to input-1 spikes (Fig. 5(b), see also Fig. 7), and the two inputs are completely independent. (The reason that the SI cell does not fire in response to input-2 spikes will be explained shortly.)

In order to get some insight into the mechanism behind this phenomenon, we look at the probability of the cells firing right after an input pulse, as a function of the other input’s phase (Fig. 7). For the FS cell, the results are what we expect: it always fires when it is excited by either input, except if the phase of the other input is small, implying that the FS cell has very recently fired in response to that input (and hence is still inhibited by its autapse—see Sect. 3). For the SI cell, there are a few interesting observations to make: first, the cell never fires in response to input-2; second, there is a considerable amount of time at the beginning of the input-2 cycle during which the SI cell does not fire in response to input-1, despite the fact that it has not fired at the beginning of the cycle (in response to input-2); and third, the SI cell also never fires in response to input-1 for large phases of input-2, meaning in the later part of the input-2 cycle. Finally, the SI cell fires on *every* input-1 pulse that falls inside the interval of input-2 phases that it is allowed to fire (which can be surprising at first sight, since it cannot fire on consecutive input-1 cycles and the two inputs are unrelated).

The first two of these observations can be easily explained as follows: due to the different dynamics of the two cells, as well as the larger rise time constant of the input-2→SI synapse compared to the input-2→FS synapse (IB→SI and IB→FS synapses in the initial network—see Tables 3, 5), when an input-2 pulse arrives, the FS cell fires first and inhibits the SI cell, preventing it from firing. This inhibition effectively lasts about 16 ms and it accounts for the lack of SI spikes both in response to input-2 (Fig. 7(b)) and near the beginning of the input-2 cycle, in response to input-1 (Fig. 7(a)). The reason why there are no SI spikes near the *end* of the input-2 cycle (Fig. 7(a)) is more complex and will be answered in Sect. 4.

#### Robustness to input timing variation

So far we have been considering inputs that are exactly periodic, meaning that every input pulse is separated from the previous one by exactly the same amount of time. But oscillations in the brain are rarely that regular [22]. It is therefore important to check that these results are robust to noise in the input timing.

**Figure 8:**
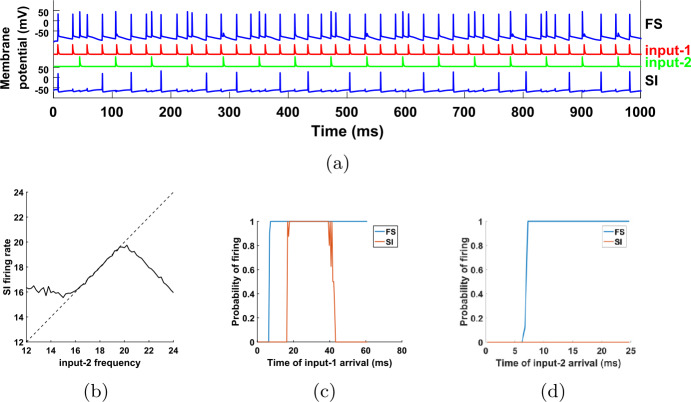
The relations between the SI cell and the inputs continue to hold even if the inputs are not exactly periodic. Compared to Figs. 5(b), 6 and 7, the interpulse intervals for each input are now random, independent of one another. Their distribution is chosen to be normal with mean equal to the nominal period (here 40 Hz for input-1 and 16.357 Hz for input-2), and standard deviation equal to 5% of the mean. (**a**) The SI cell still fires in phase with input-1, at a firing rate equal to that of input-2. (**b**) As we vary the input-2 frequency, the SI firing rate follows closely. Dashed line: diagonal. (**c**), (**d**) The FS cell fires in response to either input, while the SI cell fires only in response to input-1 pulses, and as long as these pulses arrive inside an interval of time after an input-2 pulse (see also caption of Fig. 7)

Another reason why we may consider deviations of the input from a purely periodic signal is because information might be encoded in the timing of the pulses [23–25]. Since the SI cell in our network fires in phase with input-1, it can relay information encoded in the timing of this input (see Discussion for an application to signal processing and time-division multiplexing). But again, for this we would need to make sure that the SI cell fires in phase with input-1 even when the latter is not exactly periodic. Figure 8 shows that our results continue to hold even if the input periods deviate from the nominal value by about 5%. The amount of robustness naturally depends on the parameters of the model. The analysis of Sect. 4, in particular Theorem 4.1, gives a bound on the variability of input-2 that is a sufficient condition for the results to hold, if input-1 is exactly periodic.

#### SI cell with two inputs

**Figure 9:**
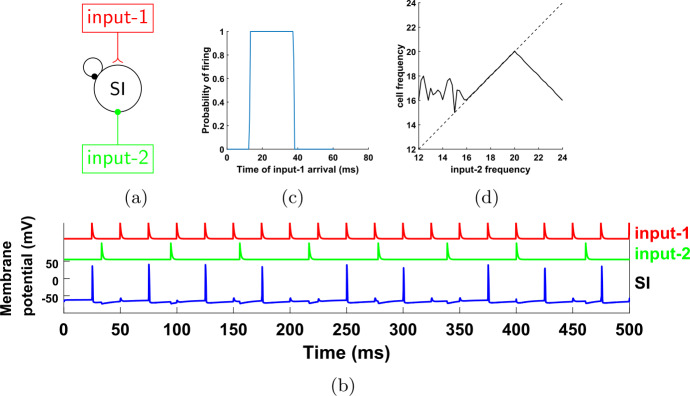
A simpler model can exhibit similar behavior to that of the FS-SI network. (**a**) Network schematic. The SI cell and input-1 are identical to the ones in Fig. 5(a). Input-2 is now inhibitory: the synapse from input-2 onto SI has the dynamics of the FS→SI synapse of the network in Fig. 5(a). (**b**) A simulation of the network in (**a**). Input frequencies are the same as in Fig. 5(b). The SI cell behaves as in Fig. 5(b), firing in phase with input-1, alternately with input-2. (**c**) Probability of the SI cell firing immediately after an input-1 pulse, as a function of the input-2 phase (time from last input-2 pulse). As in Fig. 7(a), the SI cell fires immediately after an input-1 pulse, if and only if the time since the last input-2 pulse lies in an interval of length 25 ms. (**d**) Firing rate of the SI cell as a function of the input-2 frequency. As in Fig. 6, the cell’s frequency follows that of input-2, for a range of frequencies. Small quantitative differences with the FS-SI network are due to differences in the spike form of input-2 compared to the FS cell, and to the lack of the excitatory synapse from input-2. Dashed line in (**d**): diagonal

Recall from the discussion in Sect. 3.4.1 that the FS cell fires in response to input-2 and it inhibits the SI cell, thus preventing the latter from firing in response to the excitation it gets at the same time from input-2, as well as in response to any input-1 pulses for about 16 ms. But it does not prevent the SI cell from firing in response to input-1 otherwise. In other words, from the point of view of the SI cell, the role of the FS cell is only to effectively convert the excitatory input-2 into an inhibitory input for the SI cell. This suggests a further simplification in our network, if we want to focus on the SI cell: removing the FS cell and making input-2 an inhibitory input, as shown in Fig. 9(a). Since the inhibition was previously mediated by the FS→SI synapse, in this network we have also changed the dynamics of the input-2→SI synapse to mimic the dynamics of the FS→SI synapse in the initial network (see Appendix D.1). Note that this means that we are ignoring completely the excitatory effect of the input-2→SI synapse that was present in the network of Fig. 5.

Figure 9(b) shows a simulation of the single-cell network of Fig. 9(a). Figure 9(c) and (d) are analogous to Figs. 7(a) and 6, respectively. All the essential features of the SI cell behavior are the same as before, in particular the fact that it fires only inside an interval of input-2 phases and for a range of input-2 frequencies the SI firing rate is equal to this frequency. Thus, the mechanism that allows this behavior is still present in this simpler network.

### Abstract model

Let us review what observations in the behavior of the SI cell we have and what we have not been able to explain so far. Based on the network topology and the dynamics of the system, we have been able to explain the following properties of the SI cell:

1. When it fires, it fires in phase with input-1.
2. It does not fire at two consecutive input-1 pulses.
3. It does not fire near the beginning of the input-2 period.

The properties that do not immediately follow are the following:

4. It does not fire near the end of the input-2 period.
5. It fires on every input-1 pulse that falls in the “allowed” interval of input-2 phases.
6. It fires exactly once per input-2 period.

Is it possible that properties 4–6 are consequences of properties 1–3 (perhaps together with some quantitative restrictions on the parameters)? If that were the case, then we should be able to reproduce observations 4–6 in an idealized model that is built to explicitly satisfy properties 1–3 and nothing more. Note, however, that properties 2 and 3 do not specify exactly on which input-1 pulses the cell fires; they merely give conditions under which the cell is prevented from firing. We will thus make the following stronger assumption:

7. The SI cell fires on *every* input-1 pulse that does not fall in the cases of properties 2 and 3.

The abstract model will thus be as follows: Input-1 pulses occur at integer multiples of $T_{1}$ and input-2 pulses occur at integer multiples of $T_{2}$ (where $T_{1}$ and $T_{2}$ correspond to the periods of the two inputs). For each input-1 pulse (multiple of $T_{1}$), we say that “the cell fires” if it did not fire in the previous input-1 pulse *and* the time elapsed from the last input-2 pulse is at least equal to some constant *c*, which corresponds to the effective time of inhibition from input-2 (see property 3).

**Figure 10:**
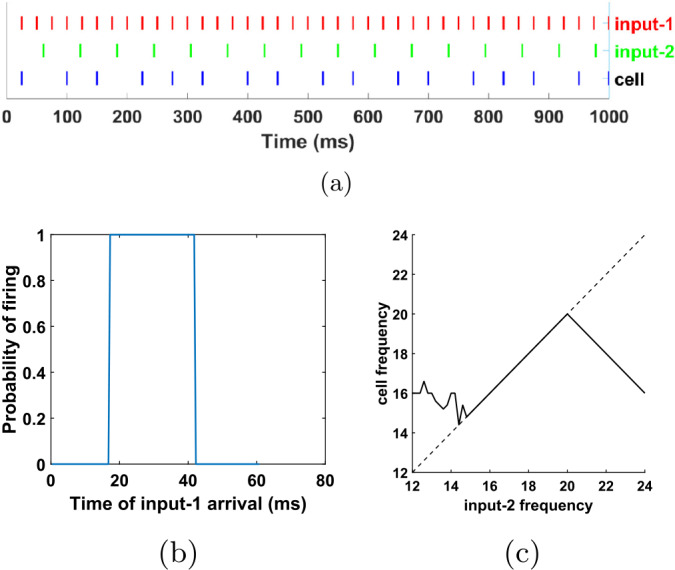
An abstract mathematical model that reproduces the behavior of the SI cell. (**a**) The red lines mark multiples of $T_{1}=25$ ms (frequency 40 Hz), while green lines mark multiples of $T_{2}=61.14$ ms (16.357 Hz). At each red line we say that the hypothetical cell fires (and draw a blue line) if it did not fire on the previous red line and the time elapsed from the last green line is at least $c=17$ ms. Such a system by construction satisfies conditions 1–3 and 7 of Sect. 3.5. It turns out that it also satisfies properties 4–6, as shown here and in (**b**). (**b**) Probability of the cell firing immediately after an input-1 pulse, as a function of the input-2 phase (time from last input-2 pulse). As in Figs. 7(a) and 9(c), the cell fires immediately after an input-1 pulse, if and only if the time since the last input-2 pulse lies in an interval of length 25 ms. (**c**) Firing rate of the cell as a function of the input-2 frequency. As in Figs. 6 and 9(d), the cell’s frequency follows that of input-2, for a range of frequencies. Dashed line in (**c**): diagonal

Figure 10 confirms what was suggested in the previous paragraph: for the chosen values of $f_{1}=1/T_{1}$ and *c* and for an interval of values for $f_{2}$, this abstract model also satisfies properties 4–6. We thus suggest that there is no extra property in the dynamics of the cell, the synapses, or the inputs that is required for properties 4–6; as long as properties 1–3, assumption 7, and perhaps certain restrictions on $f_{1},f_{2}$ and *c* are satisfied, then properties 4–6 should also be satisfied. In the next section we show that this is the case. We also note that conditions 1–3 and 7 can be easily satisfied by a leaky integrate-and-fire (IF) neuron with an inhibitory autapse and two inputs. Therefore, we should be able to reproduce the observed behavior with an IF neuron in place of the SI cell. This is demonstrated in Appendix C.

## Mathematical results on SI firing pattern mechanism

In this section we give mathematical results regarding the behavior of the SI cell. In particular we shall demonstrate what was suggested near the end of the last section: that properties 4–6 in Fig. 10 follow from properties 1–3 and 7, given certain restrictions on the system and input parameters.

### Main results on SI firing pattern

Let us begin with a semi-rigorous argument of why the SI cell fires only in an interval of input-2 phases. The restrictions we need on $T_{1}$, $T_{2}$ and *c* are that $T_{2}>2T_{1}$ and $c\in [T_{2}-2T_{1},T_{2}-T_{1})$. For example, if $T_{2}=65\text{ ms}$ and $T_{1}=25\text{ ms}$, then $c\in [15,40)$. Note that this condition is satisfied in the simulations of Sects. 3.4 and 3.5.

**Figure 11:**
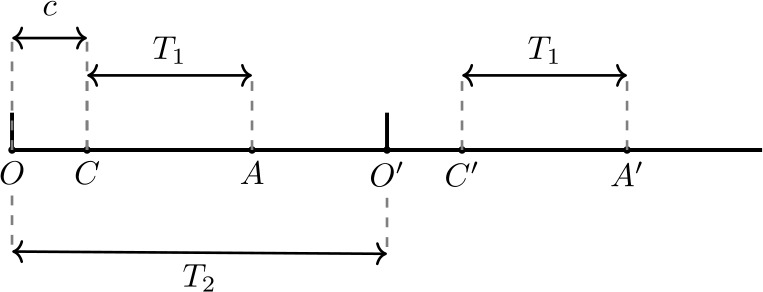
Illustration of the reason for the SI firing pattern. The figure shows two periods of input-2, with pulses coming in at *O* and $O'$. The SI cell is inhibited and cannot fire in the interval *OC* (and $O'C'$), of length *c*. Point *A* lies a distance $T_{1}$ to the right of *C*, while points $C'$ and $A'$ are the images of *C* and *A* in the second period. Suppose the SI cell fires at a phase inside the interval *CA*. Since at least $2T_{1}$ time must pass until it can fire again, and by assumption $CO'$ is less than $2T_{1}$, the SI cell cannot fire again in the same period. It also cannot fire in the interval $O'C'$, so it will fire again at the first input-1 pulse after $C'$. Since successive input-1 pulses are separated by $T_{1}$, the next SI spike will necessarily fall in the interval $C'A'$. This demonstrates that if the SI cell fires at a phase between *C* and *A* in one period, it will also do so in all subsequent periods

The mechanism can be easily explained in terms of the phase of input-2 at successive SI cell spikes. For convenience, let us measure input-2 phase in time units, from 0 to $T_{2}$. Suppose that an SI spike occurs at input-2 phase between *c* and $c+T_{1}$ (see Fig. 11). When is the next possible time for the SI cell to fire? Certainly not in the same input-2 period, since $T_{2}< c+2T_{1}$ and the SI spikes have to be separated by at least $2T_{1}$. Neither is it possible for it to fire between 0 and *c* of the next input-2 period. The first possible time for the SI cell to fire again is at the first input-1 pulse after *c*, i.e. the unique input-1 pulse that falls between *c* and $c+T_{1}$ in the next input-2 period. It will fire as long as at least two input-1 cycles have passed from the previous spike, which is guaranteed by the fact that $T_{2}>2T_{1}$. In other words, if the SI cell fires at input-2 phase between *c* and $c+T_{1}$, then it will fire again at the unique input-1 pulse that falls between phase *c* and $c+T_{1}$ in the next input-2 period.

Notice that the above conclusion (proven in a more general setting as Theorem 4.1) and a simple inductive argument easily explains properties 4–6 of the SI cell from Sect. 3.5, as long as there is at least one SI spike at an input-2 phase between $[c,c+T_{1})$. It turns out (see Theorem 4.1) that this occurs after at most $K= \lceil \frac{T_{2}-(c+T_{1})}{T_{2}-2T_{1}} \rceil +1$ input-2 cycles after both inputs are active, where $\lceil \cdot \rceil $ denotes the ceiling function. For the values of $T_{1}$, $T_{2}$, and *c* in the simulations of Sect. 3.4 (25, 61.13, and 16 ms, respectively), we get $K=3$. This implies that the transient interval is short, which is also evident in all of the simulations above. In Theorem 4.1 it is also shown that the SI cell fires exactly once per input-2 period (regardless of input-2 phase) as soon as both inputs have been activated. This can also be seen to be true in our simulations (Figs. 5(b), 8 and 9(b)).

The version of the problem considered in Theorem 4.1 is more general in two ways: first, instead of assuming that at least two input-1 cycles have to pass between successive spikes of the SI cell, we generalize this and say that there is some $m\in \{ 2,3,\ldots \} $, such that at least *m* input-1 cycles have to pass between successive SI spikes; second, we do not assume that input-2 is strictly periodic, but we allow the interpulse interval to vary from cycle to cycle. Note that the last point is an important generalization, because in the initial network the IB cell was not exactly periodic.

Let us denote by $s_{n}$, $t_{n}$, and $u_{n}$ the time of the *n*th input-1 pulse, *n*th input-2 pulse, and *n*th SI spike, respectively. We do not assume anything about when the two inputs begin relative to each other, but our results refer to intervals of time after both inputs have started. We choose an arbitrary SI spike (occurring at some input-1 pulse and after input-2 has started) to give the index 1 ($u_{1}$). We index the input-1 pulses so that $s_{1}=u_{1}$ and the input-2 pulses so that $u_{1}\in [t_{1},t_{2})$. Note that, since SI spikes do not occur in $[t_{1},t_{1}+c)$, we automatically get $u_{1}\in [t_{1}+c,t_{2})$. Without loss of generality, we set $t_{1}=0$.

Since we do not assume the intervals between input-2 pulses to be constant, there is no such thing as $T_{2}$, but an interpulse interval $t_{n+1}-t_{n}$ for each *n*. Thus the assumption $T_{2}>2T_{1}$ is modified to $t_{n+1}-t_{n}>m\cdot T_{1}$ for each $n\in \mathbb{N}$ (recall that *m* is the minimum number of input-1 cycles between successive SI spikes). Also, the assumption $c\in [T_{2}-2T_{1},T_{2}-T_{1})$ now becomes

8 $$ \sup_{n\in \mathbb{N}} \{ t_{n+1}-t_{n}\}-m\cdot T_{1} \leq c< \inf_{n\in \mathbb{N}} \{ t_{n+1}-t_{n} \}-T_{1}. $$

Note that for such a *c* to exist, we must have

9 $$ \sup_{n\in \mathbb{N}} \{ t_{n+1}-t_{n} \}- \inf_{n\in \mathbb{N}} \{ t_{n+1}-t_{n} \}< (m-1) \cdot T_{1}, $$

which poses a bound for the variability of the input-2 period.

For input-1, since we are assuming that it is periodic with period $T_{1}$, we have $s_{n}=s_{1}+(n-1)\cdot T_{1}$ (with $s_{1}$ as defined above). To describe the times of the SI spikes $u_{n}$, it will be useful to introduce some notation for the “time since the last input-2 spike”. For $x\geq 0$ we define

10 $$ \overline{x}=x-\max_{n\in \mathbb{N}}\{ t_{n}:t_{n}\leq x \}. $$

Then, for $n\geq 2$, $u_{n}$ is equal to the smallest $s_{l}$ that is larger than or equal to $u_{n-1}+m\cdot T_{1}$ and such that $\overline{s_{l}}\geq c$. We now state the theorem.

#### Theorem 4.1

*Let* $m\in \mathbb{N}$ *with* $m\geq 2$, $T_{1},c>0$, *and* $\{t_{n}\} $ *a sequence with* $t_{1}=0$ *and satisfying*

11 $$ \begin{aligned} &\inf_{n\in \mathbb{N}} \{ t_{n+1}-t_{n}\} >\max \{m \cdot T_{1},c+T_{1} \}\quad\textit{and} \\ &\sup_{n\in \mathbb{N}} \{ t_{n+1}-t_{n} \} \leq m \cdot T_{1}+c. \end{aligned} $$

*Let* $u_{1}=s_{1}\in [c,t_{2})$ *and for* $n\geq 2$ *define* $s_{n}=s_{1}+(n-1)\cdot T_{1}$ *and*

12 $$ u_{n}=\min_{l\in \mathbb{N}} \{ s_{l}:s_{l} \geq u_{n-1}+m \cdot T_{1} \textit{ and } \overline{s_{l}} \geq c\}. $$

*Also define*

13 $$ K= \biggl\lceil \frac{\sup_{n\in \mathbb{N}} \{ t_{n+1}-t_{n}\}-(c+T_{1})}{\inf_{n\in \mathbb{N}} \{t_{n+1}-t_{n}\}-m\cdot T_{1}} \biggr\rceil +1. $$

*We have the following*:

- *For any* $n\in \mathbb{N}$, $u_{n}\in [ t_{n}+c,t_{n+1} ) $.
- *For any* $n\geq K$, $\overline{u_{n}}\in [c,c+T_{1})$.
- *For any* $l\in \mathbb{N}$ *such that* $s_{l}\geq t_{K}$, $s_{l}$ *is equal to some* $u_{n}$ *if and only if* $\overline{s_{l}}\in [c,c+T_{1})$.

#### Remark 4.2

Using Eq. (9), we get

14 $$\begin{aligned} K & \leq \biggl\lceil \frac{\inf_{n\in \mathbb{N}} \{ t_{n+1}-t_{n}\}-c+(m-2)\cdot T_{1}}{\inf_{n\in \mathbb{N}} \{t_{n+1}-t_{n}\}-m\cdot T_{1}} \biggr\rceil +1 \\ & = \biggl\lceil \frac{2\cdot \inf_{n\in \mathbb{N}} \{ t_{n+1}-t_{n}\}-c-2\cdot T_{1}}{\inf_{n\in \mathbb{N}}\{t_{n+1}-t_{n}\}-m\cdot T_{1}} \biggr\rceil \\ & \leq \biggl\lceil \frac{2}{1-\frac{m\cdot T_{1}}{\inf_{n\in \mathbb{N}} \{t_{n+1}-t_{n}\}}} \biggr\rceil . \end{aligned}$$

Hence, intuitively, *K* is not too large as long as $\inf_{n\in \mathbb{N}} \{t_{n+1}-t_{n}\}$ is not too close to $m\cdot T_{1}$.

In terms of our network, part (a) of Theorem 4.1 says that there is one SI spike per input-2 period. Part (b) says that after some initial input-2 periods, all SI spikes fall at input-2 phase between *c* and $c+T_{1}$. Part (c) says that (after a few initial input-2 periods) the SI cell fires in response to an input-1 pulse if and only if this pulse falls at an input-2 phase between *c* and $c+T_{1}$. These results correspond 1–1 to properties 4–6 of Fig. 10.

We prove Theorem 4.1 in Appendix A. In Appendix B we give an application to a number-theoretic result related to Weyl’s equidistribution theorem [26, pp. 105–113].

### Allowed range for $f_{1}$ and $f_{2}$

As noted in the previous section, allowing the input-2 interpulse intervals to have a varying size is important for applying the results of the previous section to the initial network, where the IB cell was not exactly periodic (see Sect. 5.1). But the version of the network with two inputs into one cell has some interest on its own as well. Among other things, it shows how a cell can be made to fire in phase with one input, but with its firing rate controlled by another, independent input. Here we take a closer look at the range of frequencies of the two inputs for which the conditions of Theorem 4.1 hold. To do this, we will assume that input-2 is periodic, just like input-1, meaning that $t_{n+1}-t_{n}$ is constant, equal to some $T_{2}>0$. In this case, Theorem 4.1 gives sufficient conditions, in terms of *m*, *c*, $T_{1}$, and $T_{2}$, for the SI cell to fire once per input-2 cycle (Eq. (11)), and in particular to have a frequency (average firing rate) equal to $f_{2}=\frac{1}{T_{2}}$. We can recast Eq. (11) in terms of the frequencies of the two inputs to get

15 $$ \frac{f_{1}}{f_{1}\cdot c+m}\leq f_{2}< \min \biggl\{ \frac{f_{1}}{m}, \frac{f_{1}}{f_{1}\cdot c+1} \biggr\} . $$

We note that neither *m* nor *c* appear explicitly as parameters in our model, nor can they be analytically calculated; however, they can be determined from the simulation results. For example, for the parameters used in Fig. 5(b), we clearly see that the SI cell spikes are sometimes separated by two input-1 cycles, but never by less than two cycles, so *m* (the minimum number of input-1 cycles that must separate any two SI spikes) is equal to 2. Also, from Fig. 7(a) we see that the SI cell does not fire in the first 16 ms of the input-2 cycle, so $c\approx 0.016$ (in seconds). For these values of *m* and *c*, and with $f_{1}=40\text{ Hz}$, Eq. (15) becomes

16 $$ 15.15\approx \frac{20}{20\cdot 0.016+2}\leq f_{2}< 20. $$

**Figure 12:**
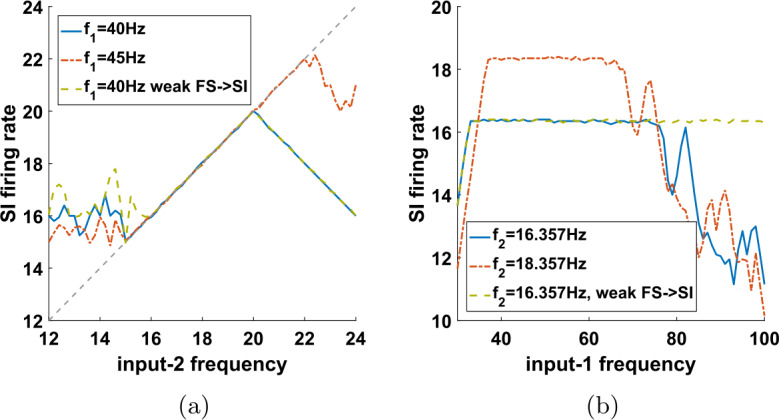
Numerical results for the range of validity of $f_{SI}=f_{2}$. (**a**) As in Fig. 6, we plot the firing rate of the SI cell as a function of the input-2 frequency $f_{2}$, but for a variety of other parameter values. The range of $f_{2}$ for which Theorem 4.1 guarantees that $f_{SI}=f_{2}$ is given by Eq. (15). For all three conditions shown in the figure (input-1 frequency 40 Hz, 45 Hz, and 40 Hz with a weaker FS→SI synapse—see Appendix D.1) we have $m=2$. Under the first two conditions we have $c\approx 0.016$ (estimated from simulations, see text), and in the third condition $c\approx 0.01325$, thus the range that Eq. (15) predicts $f_{SI}=f_{2}$ should hold is $f_{2}\in [15.15,20)$ for the first condition, $f_{2}\in [16.54,22.5)$ for the second, and $f_{2}\in [15.81,20)$ for the third condition shown. The simulation results show that in each case $f_{SI}=f_{2}$ indeed holds at least in the corresponding range, verifying the theoretical predictions. Dashed gray line: diagonal. (**b**) The SI firing rate as a function of input-1 frequency $f_{1}$. The range of $f_{1}$ for which $f_{SI}=f_{2}$ is predicted to hold is given by Eq. (17), but the parameter *m* in general depends on $f_{1}$. In all three conditions shown here (input-2 frequency 16.357 Hz, 18.357 Hz, and 16.357 Hz with a weaker FS→SI synapse—see Appendix D.1) the equality $f_{SI}=f_{2}$ holds for a large range of input-1 frequencies

Figure 12(a) demonstrates numerically that the conclusion of Theorem 4.1 indeed holds when $f_{2}$ lies in the predicted range for the above and other sets of parameter values. Note that no statement is made for values of $f_{2}$ outside the above range.

Similarly, we could solve Eq. (11) for $f_{1}$ to get

17 $$ \max \biggl\{ m\cdot f_{2},\frac{f_{2}}{1-c\cdot f_{2}} \biggr\} < f_{1} \leq \frac{m\cdot f_{2}}{1-c\cdot f_{2}} $$

However, note that *m* will naturally depend on $f_{1}$, because changing the frequency of input-1 will affect the number of input-1 cycles that the SI cell has to skip between its successive spikes. Specifically, we expect *m* to increase as $f_{1}$ increases, so that the actual set of frequencies $f_{1}$ for which Eq. (17) holds can be very different from the range suggested for any particular value of *m*. Figure 12(b) shows $f_{SI}$ as a function of $f_{1}$ for various choices of other parameters.

Finally, we note that none of the above conditions involves absolute bounds on the allowed frequencies. For instance, multiplying all of the parameters $f_{1}$, $f_{2}$, *m*, and *c* by the same scalar has no effect on the validity of these conditions. In other words, these results are applicable to inputs of very different frequencies, as long as there are matching mechanisms that set *m* and *c*, i.e. how often the SI cell can fire and for how much time after an input-2 pulse it cannot fire. This is important, since brain rhythms can range in frequency from less than 1 Hz to more than 100 Hz [1].

### Allowing SI to fire on every cycle

One of the assumptions of Theorem 4.1 is that $m\geq 2$, meaning that the number of input-1 cycles that have to elapse between successive SI spikes is at least two. But it says nothing about the case $m=1$, that is, if the SI cell could fire on every input-1 cycle. Of course, in this case the condition $c+T_{1}< t_{n+1}-t_{n}\leq c+m\cdot T_{1}$ cannot be satisfied. If we ignore this condition and set $m=1$, then the dynamics are qualitatively different. We show this in the case that $t_{n+1}-t_{n}$ is constant, equal to $T_{2}>0$. Note that in this case Theorem 4.1 implies that the SI firing rate is equal to $f_{2}=1/T_{2}$, since the SI cell fires once per input-2 period. In contrast, here we show that for $m=1$, the SI firing rate is $f_{1}\cdot (1-c\cdot f_{2})$. Regarding the notation introduced in Eq. (10), note that here, since $t_{n}=(n-1)\cdot T_{2}$ for any $n\in \mathbb{N}$, we see that, for any $x\geq 0$, $\overline{x}=x\mod T_{2}$.

#### Theorem 4.3

*Let* $T_{2}>T_{1}>0$, $c\in [0,T_{2})$, *and* $u_{1}=s_{1}\in [c,T_{2})$. *For* $n\geq 2$, *define* $s_{n}=(n-1)\cdot T_{1}$ *and*

18 $$ u_{n}=\min_{l\in \mathbb{N}} \{ s_{l}:s_{l}>u_{n-1} \textit{ and } \overline{s_{l}}\geq c\}. $$

*Then we have the following two cases*:

- *If* $\frac{T_{1}}{T_{2}}\notin \mathbb{Q}$, *then* $\lim _{n\to \infty }\frac{n}{u_{n}}=\frac{1}{T_{1}}\cdot ( 1-\frac{c}{T_{2}} )$.
- *If* $\frac{T_{1}}{T_{2}}=\frac{p}{q}$ *with* $p,q$ *relatively prime positive integers*, *then* $\vert \lim _{n\to \infty }\frac{n}{u_{n}}-\frac{1}{T_{1}}\cdot ( 1- \frac{c}{T_{2}} ) \vert <\frac{1}{q\cdot T_{1}}$.

**Figure 13:**
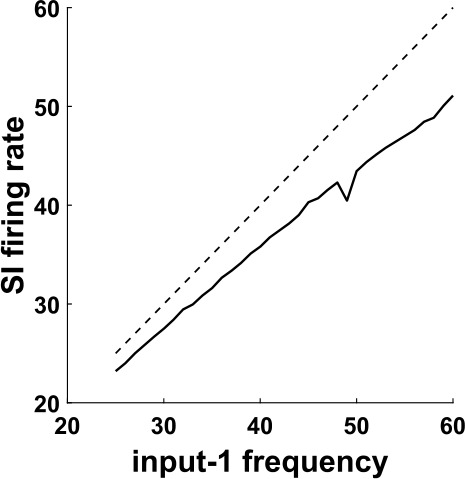
Numerical verification of the result of Theorem 4.3. Compared to the parameters used in Fig. 12(b), we have doubled the input-1 strength, so that the SI cell can now fire on every input-1 cycle. The SI firing rate is now approximately a linear function of the input-1 frequency. Dashed line: diagonal

The proof is given in Appendix A. Theorem 4.3 says that, if $f_{2}$ is kept constant, then $f_{SI}:=\lim _{n\to \infty }\frac{n}{u_{n}}$ will vary approximately linearly with $f_{1}$ (Fig. 13). This is in sharp contrast with Theorem 4.1 where, for a range of values for $f_{2}$, $f_{SI}$ did not depend on $f_{1}$ and was equal to $f_{2}$ (see also Fig. 12(b)). We therefore see that the fact that the SI cell could not fire as fast as the input-1 pulses was essential in Theorem 4.1.

## The FS-SI-IB network

### Transfer of results to the FS-SI-IB network

Our initial motivation for studying the FS-SI network of Sect. 3.4.1, which led us to the analysis of the abstract model of Sects. 3.5 and 4.1, was a question about the FS-SI-IB network of Fig. 4, namely what mechanism makes the IB and SI cells fire alternately. In the previous section, and in particular in Theorem 4.1, we answered this same question for input-2 and the SI cell in the FS-SI network. Recall that input-2 was introduced in order to mimic the effect of the IB cell in the FS-SI-IB network, so it is natural to ask whether the same mechanism as the one suggested by Theorem 4.1 is at work between the IB and SI cells in the FS-SI-IB network. In other words, is the abstract model of Sect. 3.5, 4.1 a good model of the interactions between cells in the FS-SI-IB network, if we substitute input-2 with the IB cell?

**Figure 14:**
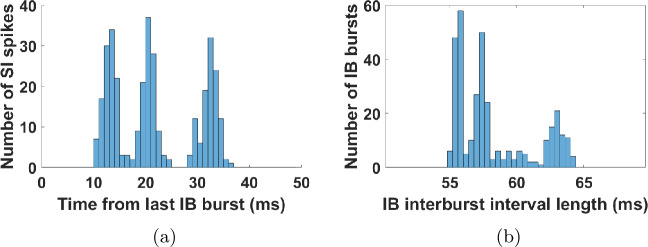
Statistics for the simulation of Fig. 4(b). (**a**) Histogram of the time elapsed from the last IB cell burst at the time when the SI cell fires. The mean IB frequency was 17.15 Hz and between any two successive IB bursts there was always a unique SI spike. The SI cell fires exclusively inside an interval of phases of the IB cell. It also fires on every input pulse (after about 1 ms) that falls in this interval (result not shown). This is similar to Fig. 7(a), with the IB cell in place of input-2. (**b**) Histogram of IB interburst interval length

What Theorem 4.1 shows is essentially that, in a system in which properties 1–3 and 7 of Sect. 3.5 hold, as well as some quantitative assumptions on the parameters given by Eq. (11), properties 4–6 will also hold. From Figs. 4 and 14(a) it is clear that, at least for the set of parameters used, analogues of properties 1–3 and 7 do hold: the SI cell, when it fires, it fires in phase with the input (within 1–2 ms); it does not fire at two consecutive input cycles; it does not fire shortly after an IB burst; and it fires at all input pulses that do not fall in the last two cases.

To check the quantitative assumptions of Eq. (11), we need first to determine the parameters *m* and *c* for the FS-SI-IB network. Similarly to what we did in Sect. 4.2, we may estimate these parameters by looking at Figs. 4(b) and 14(a). Here we get $m=2$ (minimum number of input cycles between successive SI spikes) and $c=11\text{ ms}$ (time after an IB burst that the SI cell cannot fire). Given also that $T_{1}=25\text{ ms}$, Eq. (11) becomes

19 $$ 50\text{ ms}\leq t_{n+1}-t_{n}\leq 61\text{ ms}. $$

Figure 14(b) shows that the size of the interburst interval of the IB cell is between 55 and 64 ms. Although this range is not entirely inside the bounds of Eq. (19), it is reasonably close (note that Eq. (19) is a sufficient condition, not a necessary one), so we have good reason to believe that the mechanism suggested by Theorem 4.1 is what makes the SI cell fire alternately with the IB cell.

### Non-independence of IB cell

**Figure 15:**
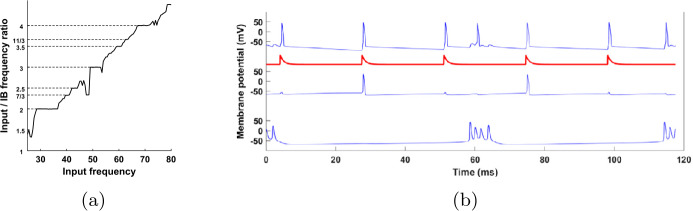
The IB firing rate depends on the input frequency. (**a**) Ratio of input frequency and IB cell firing (bursting) rate, as a function of input frequency. The IB firing rate often locks to a rational multiple of the input frequency (mode-locking). (**b**) Membrane potential of the various cells as a function of time, but time is taken modulo five input periods (here input frequency is 42.5 Hz). On each line, 34 traces are superimposed, excluding the transients. All traces fall on top of each other, showing that all cells, including the IB cell, fire periodically, with period equal to five times the input period. In both (**a**) and (**b**), the input is exactly periodic (no variation in the interpulse interval—see Sect. 2.2)

A qualitative difference between input-2 in the FS-SI network and the IB cell in the FS-SI-IB network is that input-2 is controlled externally, while the IB cell is part of the network. The success of the FS-SI network to reproduce the behavior of the SI cell relative to the IB cell, with the IB cell substituted by an externally controlled input, might lead some to believe that even in the FS-SI-IB network the interaction is mainly one-way, namely from the IB to the SI cell, while the IB cell is unaffected by the rest of the network (note that the only synaptic input the IB cell receives is from the SI cell). But this is not quite true; we have already seen that the IB cell has preferred input-1 phases for firing (Fig. 4(c)). Figure 15 shows that even the firing rate of the IB cell depends on the input frequency, with the former often mode-locking^2^ to the latter. Since in the FS-SI-IB network input-1 is the only input, in the figures and text below we refer to it simply as ‘input’.

Figure 15 raises a question: If the spike times of the SI cell are completely determined by the bursting times of the IB cell (given some fixed input), and the only possible effect of the input on the IB cell is through the SI cell, how can the IB cell mode-lock to the input? Clearly, the analysis of the FS-SI network, where the IB cell was substituted by an externally controlled input, fails to explain this. The next two sections will answer this question.

### Response of IB cell to SI spikes explains mode-locking

**Figure 16:**
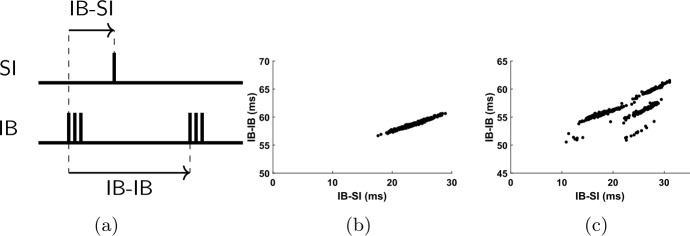
The IB burst timing depends on the SI spike timing. (**a**) Definition of variables plotted in (**b**), (**c**). IB-SI denotes the time elapsed from the beginning of an IB burst until the next SI spike. IB-IB denotes the time from the beginning of one IB burst until the beginning of the next one. (**b**) Interburst interval of the IB cell as a function of the timing of the (unique) SI spike that falls inside the interval, for a simulation of the FS-SI-IB network with 51 Hz input. The timing of the IB cell (“IB-IB”) seems to be approximately determined by the timing of the SI spike (“IB-SI”). In this simulation the SI cell fires only between approximately 16 and 30 ms. (**c**) Same as (**b**), for 62 Hz input (and slightly faster h-current kinetics—see Appendix D.1). Almost all data points fall approximately on one of three lines, suggesting that the timing of the IB cell depends on the SI timing and some other variable that takes finitely many values

Since the input effect on the IB cell is mediated by the SI cell, we can gain some insight on how the input affects the IB cell by looking at how the timing of the SI cell affects the interburst interval of the IB cell. This is shown in Fig. 16. In some cases (Fig. 16(b)), the timing of the last SI spike almost completely determines the interburst interval length of the IB cell. In other cases (Fig. 16(c)), the size of the interburst interval can take multiple values for the same SI spike timing relative to the last IB burst. We postpone the question of why we get such responses to Sect. 5.4. Here we consider the consequences of such a response, in particular how it can lead to the mode-locking seen in Fig. 15.

For convenience, in what follows we will call “IB phase” the time elapsed from the beginning of the last IB burst. Let us consider Fig. 16(b). The essential feature in this graph is that, given the IB phase when the SI cell spikes, we know the size of the interburst interval of the IB cell. Also, an increase in phase results in an increase in the interburst interval, but to a smaller extent.^3^ In the argument below we thus assume that there is some function $T_{IB}(\phi )$, defined in a suitable interval, that gives the size of the interburst interval of the IB cell when the SI cell spikes at IB phase *ϕ*. Moreover, this function is differentiable and satisfies $0<\frac{dT_{IB}}{d\phi }<1$.

We first claim that knowing the $T_{IB}(\phi )$ for a given *ϕ*, together with the input period $T_{1}$, also determines the size of the SI interspike interval. Recall that the size of the SI interspike interval is always an integer multiple of the input period $T_{1}$, so

20 $$ T_{SI}=k\cdot T_{1}, $$

where *k* is determined by $T_{1}$ and by when the IB cell fires relative to the SI cell. Note also that, if we know *ϕ* (i.e. when the SI cell fires relative to the IB cell) and consequently $T_{IB}(\phi )$ (the IB interburst interval length), then we know when the IB cell fires next, relative to the SI cell. Thus, *k* becomes a function of $T_{1}$ and *ϕ*, which allows us to write $k=k(T_{1},\phi )$ and, by Eq. (20), $T_{SI}=T_{SI}(T_{1},\phi )=T_{1}\cdot k(T_{1},\phi )$. Note also that by the analysis in Sects. 4 and 5.1, small differences in either the IB timing or the input timing do not result in differences in *k*, so $k(T_{1},\phi )$ is a step function, constant for intervals of values of its two arguments.

Suppose now that for some $T_{1}$, there is some $\phi ^{*}$ such that the periods of the IB and SI cells become equal, that is,

21 $$ T_{IB} \bigl(\phi ^{*} \bigr)=T_{SI} \bigl(T_{1},\phi ^{*} \bigr)=T_{1}\cdot k \bigl(T_{1},\phi ^{*} \bigr) .$$

For example, given the graph of Fig. 16(b), this can happen if for $T_{1}=30\text{ ms}$ and $\phi \approx 25\text{ ms}$, $k=2$. We claim that Eq. (21) implies that stable $1:k$ mode-locked solutions exist for an interval of input periods $T_{1}$.

First notice that Eq. (21) implies that there is a periodic orbit where both cells fire once per $k=k(T_{1},\phi ^{*})$ input cycles and the SI cell fires time $\phi ^{*}$ after the IB cell. Moreover, since $k(T_{1},\phi )$ is constant for small changes in both arguments (in particular continuous) and $\frac{dT_{IB}}{d\phi }\neq 0$, by the inverse function theorem we see that the above equation has a solution $\phi ^{*}(T_{1})$ for an interval of values of $T_{1}$, with the same *k*. Therefore, for an interval of values of $T_{1}$, we get a periodic pattern where both cells fire once per *k* input cycles.

But is this periodic pattern stable? For a given $T_{1}$, suppose that the SI cell fires at a phase $\phi \neq \phi ^{*}=\phi ^{*}(T_{1})$. At the next cycle it will fire at

22 $$ \phi '=\phi +T_{SI}(T_{1},\phi )-T_{IB}(\phi ). $$

As explained above, small differences in phase do not lead to different *k*, so we have $T_{SI}(T_{1},\phi )=T_{SI}(T_{1},\phi ^{*})=T_{IB}(\phi ^{*})$. Therefore,

23 $$ \phi '=\phi +T_{IB} \bigl(\phi ^{*} \bigr)-T_{IB}(\phi )\approx \phi - \frac{dT_{IB}}{d\phi }\bigg|_{\phi ^{*}} \cdot \bigl(\phi -\phi ^{*} \bigr).$$

Since $0<\frac{dT_{IB}}{d\phi } \vert _{\phi ^{*}}<1$, we see that the phase will be pushed towards equilibrium with rate $\frac{dT_{IB}}{d\phi } \vert _{\phi ^{*}}$, which shows linear stability of the solution.

To summarize, we have shown that if we have a pair $T_{1},\phi $ such that Eq. (21) holds and $0<\frac{dT_{IB}}{d\phi }<1$, then we get a range of input periods $T_{1}$ for which stable $1:k$ mode-locked solutions exist. This justifies the existence of flat parts in the graph of Fig. 15(a) at integer values of the vertical axis. A similar but more involved argument can be made for $p:q$ mode-locking with $p>1$.

#### IB cell may mode-lock without phase-locking to the input

**Figure 17:**
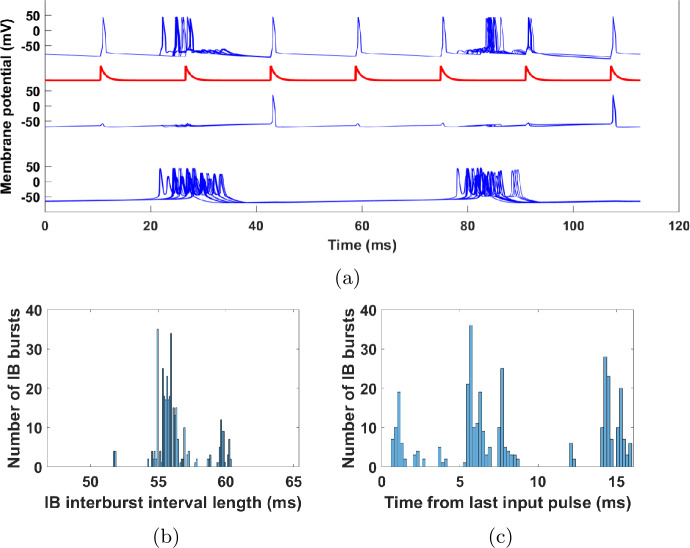
The IB cell may mode-lock without phase-locking to the input. (**a**) Membrane potential of the various cells, as a function of time modulo seven input periods (see also Fig. 15(b)). Here input frequency is 62.1 Hz. The SI cell fires periodically, twice in every seven input periods. The FS and IB cells fire only approximately periodically. There is always a single IB burst per SI interspike interval. In particular, the IB cell always bursts twice in every seven input cycles. (**b**) Histogram of IB interburst interval length. (**c**) Input phase (time from last input pulse) when the IB cell fires

Notice that in the above argument, we did not merely show mode-locking (firing once per *k* cycles), but also phase-locking (firing at a fixed input phase) of both cells. But these two phenomena do not necessarily have to occur together; Fig. 17 shows an example of mode-locking without phase-locking: both the IB and SI cells fire consistently 2 times per 7 input cycles, but without the IB cell firing at fixed phases of the input.

This can be initially surprising, since the stability argument made above (Eq. (23)) can be adapted to show that if the SI cell has a periodic firing pattern (as it does in Fig. 17(a)) and the size of the interburst interval of the IB cell is given by a map as in Fig. 16(b), then the IB cell should also converge to a periodic firing pattern with the same period. Indeed, suppose first that $T_{SI}$ is constant (i.e. we know that the SI cell interspike interval is always the same). Then from Eq. (22) and the fact that there is one SI spike per IB interburst interval it follows that

24 $$ \phi _{n+1}=f(\phi _{n}), $$

where $f:[0,a]\to [0,a]$ is defined by

25 $$ f(x)=x-T_{SI}-T_{IB}(x), $$

and *a* is a bound on the IB interburst interval length. Since we are assuming that $0<\frac{dT_{IB}}{dx}<1$, we get $-1< f'(x)<0$, hence *f* has a unique, stable fixed point $x_{0}$ that is globally attracting, and $\lim _{n\to \infty }\phi _{n}\to x_{0}$.

The above argument can be generalized to the case where $T_{SI}$ is periodic with period *p*, by considering the map $\phi _{n}\mapsto \phi _{n+p}$. Therefore, in the absence of noise, we would expect the IB phase relative to the input to converge to a finite set of values (of cardinality *p*), contrary to what is illustrated in Fig. 17.

The difference in Fig. 17 is that the relation between the SI spike timing and the length of the IB interburst interval is not of the type shown in Fig. 16(b), but like in Fig. 16(c). This means that for the same SI timing, the size of the IB interburst interval can take values that differ from each other by up to 10 ms, and which seem to align approximately with three distinct curves. As we will see in Sect. 5.4.3, small deviations from the periodic trajectory can result in ‘jumping’ to a different curve, thus the stability argument fails. This means that not only are we less likely to get a mode-locked solution, but even if we do get one, it does not have to be phase-locked.

### State reset explains response of IB cell to SI spikes

In Sect. 5.3 we saw that the fact that the size of the interburst interval of the IB cell is approximately determined by the timing of the SI spike, together with the behavior of the SI cell that we studied in detail in Sect. 4, can help to explain the fact that the two cells fire alternately and often they mode-lock to a rational multiple of the input frequency. But what is it that gives rise to the IB cell’s response in Fig. 16? In particular, why does the timing of the SI spike in a given period alone determine (perhaps up to a finite number of possibilities) the size of the IB interburst interval? This section deals with this question.

#### IB cell dynamics and interburst interval

Let *X* be the state space of the IB cell. Note that if we include the synaptic variables of the SI→IB and the $a\to d_{b}$ (IB axon to basal dendrite) synapses in the state of the IB cell, then the system evolves autonomously, except when the SI cell fires, in which case the SI cell has a very brief effect on the state variable of the SI→IB synapse. We will assume that this effect is instantaneous, so that the state of the cell may change discontinuously at the time of the SI spike, but evolves autonomously afterwards. That is, the dynamics of the IB cell between SI spikes are described by a one-parameter semi-group $\phi _{t}:X\to X$, and the effect of an SI spike is described by a map $h:X\to X$ which is applied at the time of the spike.

Let $x_{t}\in X$ denote the state of the IB cell, at time *t* *after a given burst* (with respect to burst initiation). If we denote by $t'$ and $t''$ the times of the first and second SI spikes after that IB burst, respectively, then

26 $$\begin{aligned} &x_{t}=\phi _{t-s}(x_{s}),\quad \text{for any }s,t, \text{ such that } 0\leq s\leq t< t', \end{aligned}$$

27 $$\begin{aligned} &x_{t}=\phi _{t-t'} \bigl(h \bigl(\phi _{t'-s}(x_{s}) \bigr) \bigr),\quad \text{for any }s,t, \text{ such that } 0\leq s< t'\leq t< t''. \end{aligned}$$

The cell will burst again when its state enters some subset $A\subset X$ of the state space. In symbols, the length *τ* of the interburst interval is

28 $$ \tau =\inf \{ t\geq t_{0}:x_{t}\in A \}, $$

where $t_{0}>0$ can be chosen to be the maximum possible duration of an IB burst, assuming that such duration is bounded. Given that the SI cell fires exactly once per interburst interval, the next IB burst occurs between the next and the second next SI spike, i.e. $t'<\tau <t''$. Let us assume that $t'$ is always larger than $t_{0}$ (recall that the SI cell does not fire for an interval after an IB burst, which in our simulations is much longer than the IB burst duration). Then we may substitute $x_{t}$ in Eq. (28) using Eq. (27) with $s=t_{0}$ to get

29 $$ \tau =\inf \bigl\{ t\geq t':\phi _{t-t'} \bigl(h \bigl(\phi _{t'-{t_{0}}}(x_{t_{0}}) \bigr) \bigr) \in A \bigr\} . $$

Note that the above expression implies that *τ* depends only on $t'$ and $x_{t_{0}}$, i.e. the IB interburst interval depends on the cell’s state at time $t_{0}$^4^ after the last burst and on the time of the next SI spike. The dependence of *τ* on $t'$ is clear in Fig. 16 (note that $t'$ is the variable on the horizontal axis and *τ* the variable on the vertical axis). But the dependence on $x_{t_{0}}$ is not obvious, at least in Fig. 16(b), since knowledge of the time $t'$ seems to almost completely determine *τ*. In what follows we argue that the reason for this surprising observation is the fact that for every IB interburst interval, $x_{t_{0}}$ always takes approximately either the same (Fig. 16(b)) or one of a small number of values (Fig. 16(c)).

#### State reset after a burst

**Figure 18:**
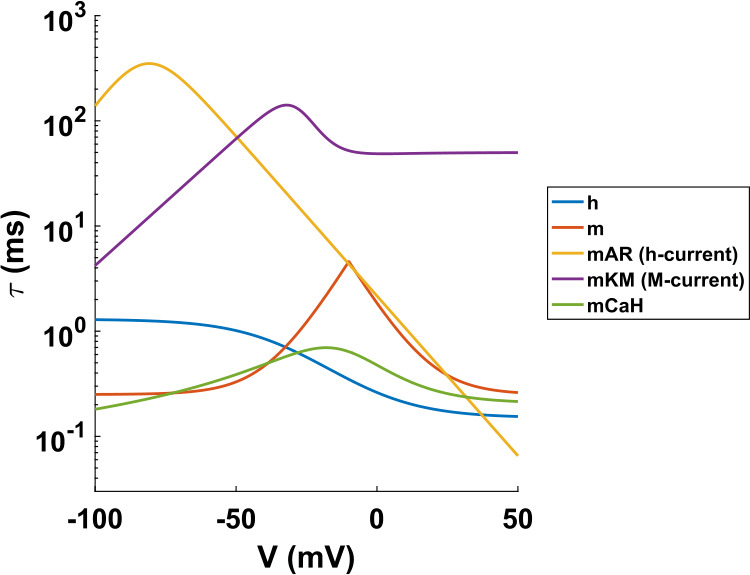
Time constants of the gating variables for the various currents of the IB cell, as functions of the membrane potential **V** (see Sect. 2.2 and Appendix D.1). For membrane potentials between −75 mV and −40 mV (between spikes), the M- and h-currents are at least 10 times slower than the rest of the currents. The $m_{KM}$ variable for the axon compartment has slightly smaller time constant than what is shown here (see Appendix D.1)

In what follows it will be useful to distinguish between slow and fast state variables. In total, the four compartments of the IB cell, together with the $a\to d_{b}$ (axon to basal dendrite) and SI→ IB synapses, involve 21 state variables (see Sects. 2.1, 2.2, Appendix D.1). The time constants of the membrane potential variables (**V**) are in the order of 1 ms. All other state variables have time constants that depend on the membrane potential of the corresponding compartment (Fig. 18). Despite this dependence on the membrane potentials, the variables can be clearly separated into “fast” (time constants of about 1 ms or less) and “slow” (time constants of $>10\text{ ms}$), at least for subthreshold values of the membrane potentials ($<-40$ mV), that is, when the cell is not firing. According to this separation, the membrane potential variables can be considered fast, while the synaptic state variables are slow, at least when the cell is not firing (time constants of 20 ms and 100 ms for the SI→IB and $a\to d_{b}$ synapses, respectively (Table 3)).

Since we are interested here in studying the size of the interburst interval of the cell, which is in the order of 60 ms, we may assume that the fast variables are at their (slow variable-dependent) equilibria and study the dynamics on the slow manifold [27, 28]. There are seven slow variables in total (see Sect. 2.2, Appendix D.1): the two synaptic state variables, two h-current gating variables (one for each dendritic compartment), and three M-current gating variables (one for each dendrite and one for the axon).^5^

**Figure 19:**
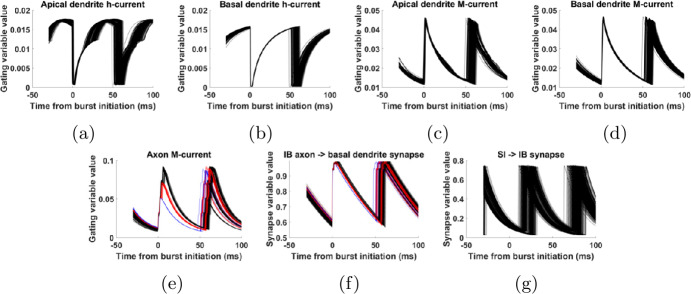
Reset of slow state variables of IB cell at bursting. (**a**) Apical dendrite h-current gating variable. Each trace corresponds to a burst and is centered so that 0 on the time axis corresponds to the initiation of the burst. The h-current gating variable (vertical axis) drops to approximately 0 at the initiation of each burst (0 on the horizontal axis). (**b**) Same as (**a**), for basal dendrite. This variable also resets to approximately 0 at each burst. (**c**), (**d**) Same as (**a**), (**b**), but for the M-current gating variables. These variable also reset to approximately fixed numbers when the cell begins to burst, around 0.045 in both cases. (**e**) Same as (**c**), (**d**), but for the axon compartment. The traces are color-coded according to the number of spikes in the burst (blue: 2 spikes, red: 3 spikes, black: 4 spikes). The M-current gating variable resets to one of three values (approximately 0.05, 0.07, or 0.09) after a burst, depending on the number of spikes in the burst. (**f**) Same as (**e**), but for the state variable of the IB $a\to d_{b}$ (axon to basal dendrite) synapse. This variable resets to approximately 1 after each burst, but the time it retains this value is variable, depending on the number of spikes in the burst. The resulting differences in the value at a given time after the burst are small. (**g**) Same as (**a**)–(**e**), but for the SI→IB synaptic state variable. This variable does not reset when the IB cell bursts, but its value is always between 0.1 and 0.2 at the beginning of a burst. Simulation parameters are as in Fig. 16(c)

Recall that we are interested in the trajectory of the IB cell state during an interburst interval. Figure 19 shows the trajectories of the slow variables, centered at the time of initiation of bursts. We see that the state variables associated with the h-current reset their value to near 0 at the beginning of a burst (Figs. 19(a) and (b)). This happens because, when the cell is spiking (membrane potential $>-40$ mV), the time constants of these variables become very small, as shown in Fig. 18, and at the same time their equilibrium is very close to 0 (see Sect. 2.2, Appendix D.1). As a result, after every spike the state variables associated with the h-current reset to approximately 0. We note that these properties of the dynamics of the h-current, also present in previous models [12, 29], agree with experimental data [30].

We also see that the state variables associated with the dendritic M-currents before the burst initiation have values around 0.01 and after each burst they increase by an approximately fixed amount, reaching a value around 0.05 (Figs. 19(c) and (d)). Although the time constants of these variables do not get smaller during a spike, their equilibrium increases towards 1 as the membrane potential increases, which results in a fast increase in their values, relative to the typical values of these variables. Moreover, the fact that spikes have a stereotypical form means that the increase in the values of these variables will be approximately the same for every spike. Combined with the fact that before the dendritic spike (at the burst initiation) the values of these variables vary little in absolute terms (in the range 0.01-0.015), they reach approximately fixed values immediately after any spike (approximately 0.05).

Recall that when the IB cell bursts, it is the axonal compartment that fires multiple action potentials, while the other compartments fire single action potentials (Fig. 3(b)). As a result, the gating variables of the dendritic M-currents increase by a single step, always to approximately the same value (Figs. 19(c) and (d)), while that of the axonal M-current increases in several steps at every burst (Fig. 19(e)), one step per spike in that burst. This means that at the end of the burst the state variable that corresponds to the axonal M-current may be at one of several values, depending on the number of spikes in that burst. A similar pattern, but less pronounced, is evident for the synaptic state variable of the $a\to d_{b}$ synapse (Fig. 19(f)). Although the value of this variable always increases to 1 when the cell bursts, it might stay there longer, depending on the number of spikes in the burst, and this leads to small differences in the value of this variable at a fixed amount of time after the burst initiation. Finally, the SI→IB synaptic state variable, although it does not reset when the IB cell bursts, its value at burst initiation is always between 0.1 and 0.2 (Fig. 19(g)). This small variation in value does not seem to affect the dynamics of the cell, since all other state variables follow approximately the same trajectory after each burst, at least until the SI→IB is activated again and its value reset to approximately 0.75.

To summarize, at a fixed amount of time $t_{0}$ after burst initiation (large enough so that the burst is guaranteed to have terminated, but before the next SI spike), the values of most slow state variables are forced to take approximately a given value, while the axonal M-current and the $a\to d_{b}$ synaptic state variable may take approximately one of a few values, depending on the number of spikes in the burst. Since the fast state variables quickly reach their new equilibria after a burst, we conclude that the full state $x_{t_{0}}$ of the IB cell at time $t_{0}$ after a burst may take (approximately) one of a few possible values, one for each possible number of spikes in a burst.

**Figure 20:**
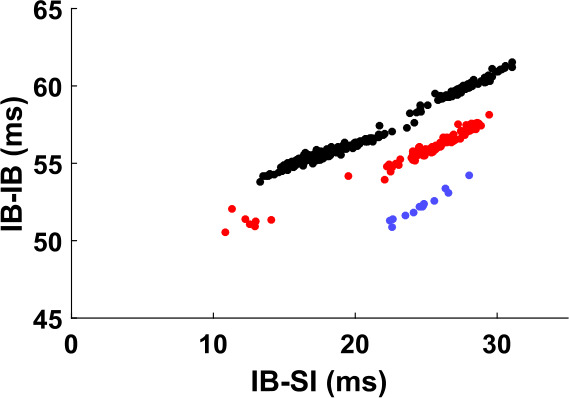
Scatterplot of IB interburst interval vs timing of SI cell, as in Fig. 16(c), but color-coded according to the number of spikes in the last IB burst (blue is for 2 spikes, red for 3 spikes, black for 4 spikes). The timing of the SI spike (“IB-SI”) combined with the number of spikes in a burst determine the length of the interburst interval (“IB-IB”)

If for some parameter values it happens that the IB cell always exhibits the same number of spikes per burst, then $x_{t_{0}}$ will take approximately the same value in every interburst interval, and by Eq. (29), for $s=t_{0}$, we see that the length *τ* of the interburst interval is a function of $t'$ only, which explains Fig. 16(b). If, on the other hand, the IB cell fires a different number of spikes in different bursts, then $x_{t_{0}}$ will take one of a few different values and, again by Eq. (29), the dependence of *τ* on $t'$ will take one of a few different forms, as is the case in Fig. 16(c). In Fig. 20 it is verified that the three different responses seen in Fig. 16(c) are associated with a different number of spikes in the last IB burst. We thus conclude that if we can predict the number of spikes in an IB burst, and we know the timing of the synaptic input it receives, we can predict the rest of the cell behavior. However, as shown in the next section, predicting the number of spikes in a burst is not possible at this level of analysis.

#### Unpredictability of number of spikes in a burst

Figure 20 shows how the number of spikes in a burst of the IB cell and the timing of the subsequent SI spike determines when the IB cell will burst again. But does it also determine the number of spikes in that next burst?

**Figure 21:**
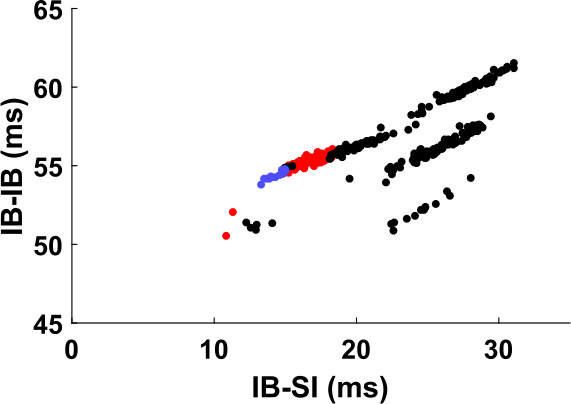
Scatterplot of IB interburst interval vs timing of SI cell, as in Fig. 20, but with the data points color-coded according to the number of spikes in the *next* burst. The number of spikes is sensitive to the timing of the SI cell. But even exact knowledge of the SI timing (“IB-SI”) and the number of spikes in the last burst (curve where the data point lies—see Fig. 20) is not always enough to determine the number of spikes in the next burst (color)

To answer this, we look at Fig. 21, which is again a plot of the length of the IB interburst interval against the SI spike timing, but this time color-coded for the number of spikes in the *next* burst. Here we see that the timing of the SI spike is not enough to predict the number of spikes in the next IB burst, even if we take into account the number of spikes in the previous burst. This suggests that the number of spikes in a burst can be very sensitive to the exact state at burst initiation (recall that the reset of the IB state variables to fixed values after a burst was only approximate—Fig. 19).

Note that differences in number of spikes in the burst in turn lead to differences in the length of the next interburst interval (Fig. 20), in the order of 5 ms, which can then lead to further differences in the number of spikes in the following burst and so on. This results in the unpredictability of the IB burst timing in the long term and explains the variability in the phase of the IB cell relative to the input in Fig. 17(c), despite the fact that its firing rate is consistently a fixed fraction of the input frequency.

### Summary of IB cell behavior

We now summarize the results of this section regarding the IB cell. The IB cell is a high-dimensional dynamical system, consisting of four Hodgkin–Huxley compartments, each with a set of state variables that describe the currents that enter or exit the cell. However, as Sect. 5.4 showed, the full state of the system is approximately reset when this cell fires, because all slow variables reset, with the notable exception of the axonal M-current gating variable, which can take one of several values immediately after the burst, depending on the number of spikes in that burst.

If the number of spikes per burst is always the same, then all state variables are reset after the burst. Consequently the cell, as a dynamical system, always follows the same trajectory after its burst ends. This trajectory is only perturbed by the unique stimulus it receives (before the next burst), that is, the synaptic input from the SI cell. Since this stimulus is always of the same form, by knowing the time that it arrives relative to the last IB burst, we know the full IB trajectory during that interburst interval, and in particular when the IB cell will burst again. We can thus create a map that describes the length of the IB interburst interval as a function of the SI stimulus timing (Fig. 16(b)). The properties of this map, combined with the fact that the SI interspike interval lengths are integer multiples of the input period, lead to stable mode-locking of the IB cell to the input (Sect. 5.3).

In the more general case, the number of spikes in an IB burst varies, and this leads to different possible states for the reset, hence different possible trajectories after the burst as well. In this case we may still observe mode-locking, which can occur even in the absence of phase-locking (Sect. 5.3.1). In contrast, when the IB cell fired the same number of spikes per burst, mode-locking always implied phase-locking (but see Discussion).

Finally, it is impossible to predict the number of spikes in the next IB burst based on the number of spikes in the previous burst and the timing of the last SI synaptic input (Fig. 21).

## Discussion

We studied the synchronization properties of a certain Hodgkin–Huxley type neuronal network, which is excited by periodic, pulsatile input. This study was motivated by the potential importance of this network for brain function and its ability to respond to an input while at the same time retaining its natural rhythm. An initial simplification of the model led us to a three-neuron network (the FS-SI-IB network), while further observations led us to consider a subset of it on its own (the FS-SI network), but with two oscillatory inputs of unrelated frequencies. The rhythms in this paper were in the beta and gamma range but, as we showed, similar results hold for other ranges of frequencies.

In the FS-SI network, we observed that the SI cell can respond to both input rhythms at the same time, but in different ways: it always fired in phase with input-1 (the faster, excitatory input), but its firing rate was equal to that of input-2 (the slower, effectively inhibitory input—Figs. 5(b) and 6). The fact that the cell fires in phase with an excitatory input is reminiscent of simple entrainment to an input [31, Ch. 3]. However, unlike the case of simple entrainment, the cell firing rate did not depend on the input-1 frequency, for a range of frequencies (Fig. 12(b)). At the same time, the fact that the SI firing rate was controlled by an inhibitory input, which prevented the cell from firing for some time after each pulse, is reminiscent of inhibition-based rhythms, in particular the well-known interneuronal gamma (ING) and pyramidal-interneuronal gamma (PING) rhythms [14]. Unlike those rhythms, however, the SI cell’s frequency was unaffected by moderate changes in the inhibition’s strength, at least in some parameter regimes (Fig. 12(b)), and was instead controlled by the input-2 frequency. Also unlike the classical inhibition-based rhythms, in our case, even when the inhibition was periodic, the phase at which the cell fired was not fixed, but uniformly distributed in an interval (Fig. 7(b)). This is an instance of mode-locking without phase-locking (unrelated to the same phenomenon seen in Sect. 5.3.1).

By making simplifying approximations, we were able to understand in detail the core mechanism that underlay the SI cell’s behavior and produce an abstract model that can reproduce all the essential features of this behavior. The fact that the necessary assumptions can be stated without reference to the actual neuronal model means that such behavior can be exhibited by other types of networks, with different types of cells, and even by non-neuronal systems. Here we demonstrated that a single-cell network (Fig. 9), even a leaky integrate-and-fire neuron (Appendix C), can reproduce the results.

The mathematical statement of the result also allowed us to see how the behavior depends on the various parameters/assumptions. For example, the fact that the SI cell was not able to fire as fast as at every input-1 cycle was essential (compare Theorems 4.1 and 4.3), while the number of cycles that it had to skip between spikes did not matter (see assumptions of Theorem 4.1). We also found the allowed ranges of input frequencies for our results to hold, as functions of other parameters (Eqs. (15) and (17)). Importantly, there are no absolute bounds in the input frequencies, except in their ratio; the same mechanism can in principle work with pairs of much higher or lower frequencies, thus it can apply to different brain rhythms, as long as other parameters are also modified appropriately, so that the stated bounds continue to hold. Also, the theoretical bounds found agreed with simulation results, verifying that the simplified mathematical description captures the essential features of the actual model (Fig. 12). As a side note, notice that, from Fig. 12(a) (and to a lesser extent from Fig. 12(b)), it seems that when the input-2 frequency is *larger than* half the input-1 frequency, the SI firing rate is equal to their difference ($f_{SI}=f_{1}-f_{2}$). However, finding the exact conditions under which this equality holds is beyond the scope of the current work. We also showed numerically that the results continued to hold even if the input was not exactly periodic, but the intervals between pulses varied randomly (Fig. 8). This is important because in a real biological neural network various sources of noise might cause an oscillatory input to deviate from its mean frequency, while in other cases information might be encoded in the timing of the input pulses [23–25].

**Figure 22:**
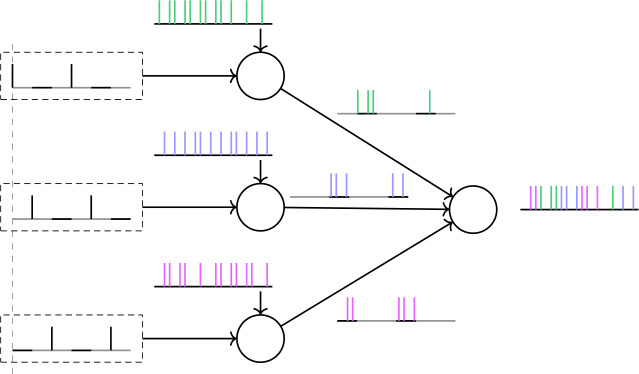
Time-division multiplexing model. Three cells each receive an information-containing input (leftmost colored bars) and a timing input (boxed traces on the left). The timing inputs given to the three cells are a shifted version of each other. They determine windows of phase (darker segments) during which the corresponding cell may echo the information-containing input; the output of the cell (colored bars in the middle) is a copy of only those spikes (of the information-containing input) that fall inside the allowed phases. All three cells send their output to a new cell that responds to all of them, echoing their spikes. As a result, its output is divided into phases, during each of which it simply repeats the information-containing input from one of the sources. Assuming that information is contained in the precise timing of the spikes, the output cell transmits information from different sources at different intervals of time

**Figure 23:**
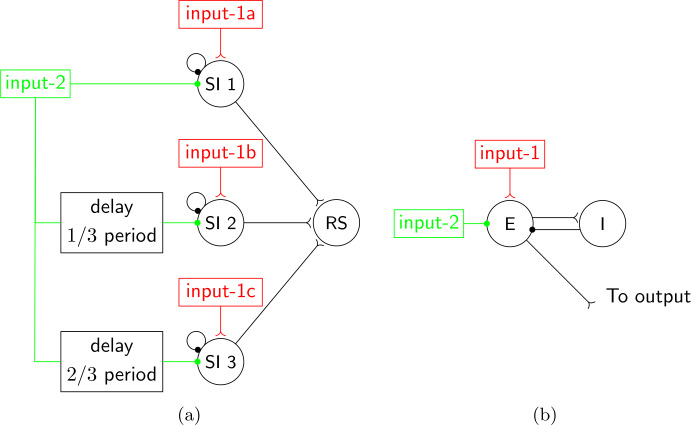
(**a**) An implementation of the network topology of Fig. 22 with three copies of the network of Fig. 9(a) and an RS cell. The three “input-1” (see also Fig. 9(a)) are independent, while the “input-2” that each SI cell is receiving is a delayed version of each other. The SI→RS synapses in this model are excitatory, contrary to the fact that the SI cells are considered inhibitory. (**b**) A more biologically realistic version of a subcomponent of the network in (**a**). To avoid having a cell with both an excitatory outgoing synapse and an inhibitory autapse (like the SI cells in (**a**)), we may consider a pair of cells, an excitatory (E) and an inhibitory (I) one. The E cell receives the inputs and provides the output, while at the same time projecting onto the I cell, and the I cell projects back to the E cell. Assuming that the I cell fires always immediately after the E cell, the pair of reciprocal connections (E→I and I→E) function effectively as self-inhibition for the E cell, playing the same role as an inhibitory autapse would. For simplicity, in the simulation of Fig. 24 we use the version of the network shown in (**a**)

**Figure 24:**
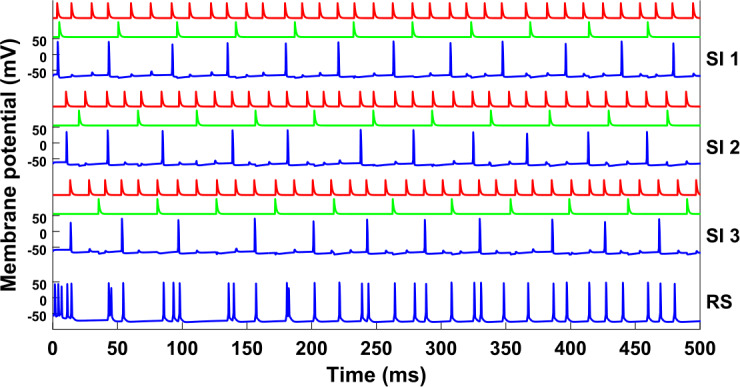
A simulation of the network in Fig. 23(a). Input-2 is 22 Hz and input-1 has average frequency 70 Hz with 10% variability in the interpulse intervals (see Appendix D.1). Above the membrane potential of each of the SI cells, the two inputs that it receives are shown (red for input-1, green for input-2). Each of the SI cells behaves as in the network of Fig. 9. The RS cell fires in response to each of the three SI cells and only to them (with the exception of the transient period). The network as a whole implements the time-division multiplexing mechanism described in Fig. 22

When information is encoded in the timing of the pulses, the properties of the SI cell also suggest a mechanism for time-division multiplexing [32, p. 123], as follows: The fact that the SI cell in our network fires in phase with input-1 means that it can relay information encoded in the timing of that input’s pulses. Moreover, since the SI cell does not fire in response to every pulse, the information that it relays will be a processed version of input-1; it will only relay certain pieces of information. In the signal-processing literature this function is called *downsampling* or *decimation* [33, pp. 167–172]. The downsampled signal that is encoded by the SI cell will include only those spikes that arrive at a certain phase of input-2 (Fig. 7(a)). In other words, input-2 is defining a window during which spikes from input-1 can be echoed and thus pass on the information encoded in their precise timing. One can then imagine that this system can form part of a time-division multiplexing mechanism; a number of sources of information of high frequency, like input-1, can be allowed to pass on information at different phases of a slower oscillation like input-2, perhaps by receiving different versions of input-2, shifted in phase from each other (Fig. 22). This information can be separated again later, if needed, either by a decoder that has access to input-2, or by having encoded the identity of the sources together with the informational content. We demonstrate the encoding mechanism by replicating the network of Fig. 9(a) three times and adding an outgoing synapse from each SI cell to a single new RS cell (Figs. 23(a) and 24). Note that in our example these outgoing synapses have to be excitatory, unlike the equations in the paper in which the SI cell is inhibitory. A more complex but possibly functionally equivalent setup with inhibition is illustrated in Fig. 23(b). This example is offered as a proof of concept that closely related networks can perform multiplexing.

In the last part of our paper we looked at the FS-SI-IB network. Compared to the FS-SI network with two inputs, the IB cell replaced the input-2, so that from the point of view of the FS-SI sub-network not much had changed, except that now it was receiving synaptic input from the IB cell, rather than the external input-2. After arguing that the results about the FS-SI network explain the corresponding part of the FS-SI-IB network behavior, we focused our attention on the dynamics of the IB cell and the way it responds to the only synaptic input that it receives, that is, from the SI cell. We showed numerically that the timing of the SI cell relative to the IB cell’s last burst, combined with the number of spikes in the last IB burst, could approximately determine the timing of the next burst of the IB cell (Fig. 20).

The plot in Fig. 20 is very similar to a phase-response curve (PRC), a widely used tool in characterizing responses of neurons to inputs [15]. A PRC gives the phase advance or delay caused by a stimulus to a periodically firing cell, as a function of the timing of that stimulus, relative to the time of the last spike of the cell. The method can also be applied to bursting cells [18]. The underlying assumption of the PRC method is that the phase advance or delay triggered by a stimulus depends *only* on the time elapsed from the last spike of the cell. In some cases this can be shown to be the case, for example if the successive stimuli are well-separated, so that the cell returns to its natural limit-cycle before the next stimulus arrives, or if the stimuli are sufficiently weak, so that the trajectory never deviates far from the limit cycle [34, ch. 8]. But in general, we should expect the “phase advance” (or delay) to depend on the full state of the cell when it receives the stimulus, rather than merely on the time since it last fired.^6^ Therefore, in a plot like Figs. 16(b) and 16(c), we would expect to see a wide distribution of data points, rather than them forming a “curve”.

The input used in Fig. 16 (SI cell synaptic input) satisfied neither of the two conditions mentioned above (weak or well-separated stimuli) that can justify a single-valued response for a given stimulus timing (Fig. 16(b)), or even a multi-valued one as in Fig. 16(c). However, a reset of the state of the IB cell every time this cell burst, effectively reduced the dependence of the cell’s state on its history (Fig. 19). This resulted in the cell being approximately at the same or at one of several possible states at a given time after its last burst, which could help explain the fact that its response was approximately a function of the stimulus timing only.

We thus see another case where a PRC-type approach can be useful, namely if the cell’s state is always the same immediately after a spike/burst, and it receives a single input (of the same form always) in every interspike/interburst interval. For the state to be always the same immediately after a spike/burst, it is enough to check that the slow variables (variables whose time constants are comparable to the length of the interspike/interburst interval or longer) take fixed values after the cell spikes/bursts. This can happen for one of three reasons: the variable resets to a fixed value at the time of the spike/burst (like the h-current state variables in the IB cell); it has a fixed value immediately before the spike/burst and remains unchanged during the spike/burst (the IB autapse state variable); or it has a fixed value immediately before spikes/bursts and changes in a predictable way during them (the M-current in the IB cell).

However, given that these resets for a high-dimensional system are usually only approximate, the description of the response through a phase-response map has its limitations. In our case, the method failed to predict the number of spikes in the IB cell’s next burst (Fig. 21). Previous studies have shown high sensitivity of the number of spikes in a burst to input timing for the Hindmarsh–Rose neuronal model [18, 19]. We emphasize, however, that in our case, even *exact* knowledge of the input timing is not enough to determine the number of spikes in the next burst. This suggests that the number of spikes in the next burst is highly sensitive to the cell’s state in a way that cannot be captured by a PRC and which is reminiscent of deterministic chaotic systems.

An extension of the PRC theory that allows dependence of the cell’s firing on more than the last stimulus is used in [35], and involves taking into account the second order PRC, which describes the effect that a stimulus has on the length of the next interburst interval, as opposed to the current one. However, the method still requires that the cell’s trajectory return to the limit cycle before the next stimulus comes in, as with the usual (first order) PRC. For the IB cell in our network, a second order PRC is unlikely to provide any additional explanatory power for the interburst interval length or the number of spikes in a burst, exactly because of the reset of the state when the cell bursts, which makes the events of the previous cycle irrelevant to the current cycle. An alternative way of characterizing the response of a neuron to non-weak, non-well-separated stimuli, is through a functional PRC (fPRC) [36]. However, this approach can only be applied when the pattern of stimulus timing is known a priori, unlike the synaptic stimulation of the IB cell in our model.

The dependence of the size of the IB interburst interval on the timing of the SI stimulus, together with the converse dependence of the SI spike timing on the synaptic input from the IB cell, was the reason for the 1–1 firing relation, and consequently the equal firing rates, of the two cells. Moreover, this firing rate was often a rational multiple of the input frequency (Fig. 15(a)), with the cells firing exactly *p* times in every *q* input cycles, a property called $p:q$ mode-locking [17]. But this mode-locking did not necessarily imply phase-locking (Fig. 17), contrary to what is often the case with neuronal models [17, 37, 38]. In our system, mode-locking without phase-locking could occur only when there were multiple possible reset states, which was the case when the number of spikes per IB burst was variable. The reason that this was impossible with a fixed number of spikes per IB burst was that, due to the dynamics of the IB cell and the SI→IB synapse, the length of the IB interburst interval increased as a function of the SI spike timing, but at a rate less than 1 (Fig. 16(b)). This translated to a map, describing the evolution of the SI spike timing relative to the IB burst, with a globally attracting fixed point (Sect. 5.3.1). But, in principle, in a different system, it is possible to have such a map where the quantity of interest remains bounded without converging to a periodic solution, as is the case with the logistic map in some parameter regimes [39, Ch. 1]. Thus, mode-locking without phase-locking should be possible even with a cell that always resets to the same state and receives a single, invariant (up to timing) stimulus per interspike/interburst interval.

To the best of the authors’ knowledge, the two phenomena of mode-locking in the absence of phase-locking that we described (IB cell in FS-SI-IB network and SI cell in the FS-SI network) are the first examples of such behavior in the neuronal dynamics literature. This is especially important, because a very common method of studying functional relations between different brain regions is to look at phase relations between oscillations exhibited at those regions [40, 41]. Synchronization in terms of phase-locking has even been suggested to modulate the ability of networks to communicate with each other [7]. Here we see that a network can drive another network in the absence of any fixed phase relations.

## Figures and Tables

**Figure 25:**
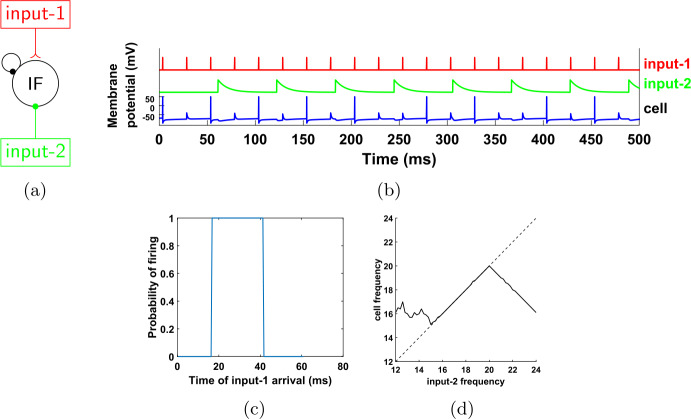
The surprising behavior of the SI cell can be reproduced by a leaky integrate-and-fire (IF) neuron. (**a**) Network schematic. The IF cell has taken the place of the SI cell in Fig. 9(a). (**b**) A simulation of the network in (**a**). Input frequencies are $f_{1}=40$ Hz and $f_{2}=16.357$ Hz. In the figure the membrane potential when the cell fires has been accentuated to 50 mV for illustrative reasons, although any time it passes the firing threshold (−40 mV) it is immediately reset to $V_{\mathrm{reset}}$ (−100 mV). The SI cell behaves as in Fig. 9(b), firing in phase with input-1, alternately with input-2 (after a short transient). (**c**) Probability of the cell firing immediately after an input-1 pulse, as a function of the input-2 phase (time from last input-2 pulse). As in Fig. 9(c), the cell fires immediately after an input-1 pulse, if and only if the time since the last input-2 pulse lies in an interval of length 25 ms. (**d**) Firing rate of the cell as a function of the input-2 frequency. As in Fig. 9(d), the cell’s frequency follows that of input-2, for a range of frequencies. Dashed line in (**d**): diagonal

## Data Availability

The code used for simulations and analysis of the results is available online [43].

## Appendix A: Proofs of theorems

Here we give the proofs of Theorems 4.1 and 4.3. For the proof of Theorem 4.1 we need the following lemma.

### Lemma A.1

*Under the assumptions of Theorem *4.1, *the following hold*:

- *For each* $n\in \mathbb{N}$, $u_{n}\in [t_{n}+c,t_{n+1})$.
- *For each* $n\in \mathbb{N}$, *at least one of the following is true*:
  - $u_{n+1}-t_{n+1}< c+T_{1}$ *or*
  - $u_{n+1}=u_{n}+m\cdot T_{1}$.
- *For each* $n\in \mathbb{N}$, *if* $u_{n}-t_{n}< c+T_{1}$, *then* $u_{n+1}-t_{n+1}< c+T_{1}$.
- *Let* *K* *be defined as in Eq*. (13). *Then*
  30 $$ u_{K}-t_{K}< c+T_{1}. $$

### Proof

- We shall use induction on *n*, the base case being true by assumption. Suppose that $t_{n}+c\leq u_{n}\leq t_{n+1}$. Since $t_{n+1}\leq t_{n}+c+m \cdot T_{1}$ (by assumption), we get
  31 $$\begin{aligned} u_{n+1} & \geq u_{n}+m\cdot T_{1} \\ & \geq u_{n}+m\cdot T_{1}-(t_{n}+c+m\cdot T_{1}-t_{n+1}) \\ & =u_{n}-t_{n}-c+t_{n+1}\geq t_{n+1}. \end{aligned}$$
  For the other direction, note first that
  32 $$\begin{aligned} u_{n}+m\cdot T_{1} & < u_{n}+m\cdot T_{1}+(t_{n+2}-t_{n+1}-m \cdot T_{1}) \\ & =u_{n}+t_{n+2}-t_{n+1}< t_{n+2}. \end{aligned}$$
  Since the interval $[t_{n+1}+c,t_{n+2})$ has length greater than $T_{1}$ (because $t_{n+2}> t_{n+1}+c+T_{1}$), Eq. (32) implies that the set $\{ k:k\geq m \text{ and } u_{n}+k\cdot T_{1}\in [t_{n+1}+c,t_{n+2}) \} $ is non-empty. Therefore, there exists some $s_{l}=u_{n}+k\cdot T_{1}$ with the properties that $s_{l}-u_{n}\geq m\cdot T_{1}$, $\overline{s_{l}}\in [c,c+T_{1})$ and $s_{l}< t_{n+2}$. Since $u_{n+1}$ is by definition the smallest of the $s_{l}$ that satisfies the first two of these properties, we get $u_{n+1}< t_{n+2}$.
- Since by assumption $t_{n+1}\leq t_{n}+c+m\cdot T_{1}$ and by part (a) $u_{n}\geq t_{n}+c$ and $u_{n+1}< t_{n+2}$, we get
  33 $$ t_{n+1}\leq u_{n}+m\cdot T_{1}\leq u_{n+1}< t_{n+2}. $$
  Therefore, if $u_{n}+m\cdot T_{1}\geq t_{n+1}+c$, then $s_{l'}=u_{n}+m\cdot T_{1}$ is the smallest $s_{l}$ with the property $s_{l}\geq u_{n}+m\cdot T_{1}$ and $\overline{s_{l}}\geq c$, hence $u_{n+1}=s_{l'}=u_{n}+m\cdot T_{1}$. Otherwise (if $u_{n}+m\cdot T_{1}< t_{n+1}+c$) $u_{n+1}$ equals the smallest number of the form $u_{n}+k\cdot T_{1}$ that is no less than $t_{n+1}+c$, i.e. the unique such number in $[t_{n+1}+c,t_{n+1}+c+T_{1})$.
- By part (b), we may assume that $u_{n+1}=u_{n}+m\cdot T_{1}$, since in the other case there is nothing to show. We have
  34 $$ u_{n+1}-t_{n+1}=u_{n}+m\cdot T_{1}-t_{n+1} \leq u_{n}-t_{n}< c+T_{1} $$
  (using the assumption that $t_{n}+m\cdot T_{1}\leq t_{n+1}$).
- If $u_{n}-t_{n}< c+T_{1}$ for some $n< K$, then the result follows from part (c). Suppose on the contrary that $u_{n}-t_{n}\geq c+T_{1}$ for all $n< K$. By part (b) we have $u_{n+1}=u_{n}+m\cdot T_{1}$, $n< K$, and in particular $u_{K}=u_{1}+(K-1)\cdot m\cdot T_{1}$, therefore
  35 $$\begin{aligned} u_{K}-t_{K} & =u_{1}+(K-1)\cdot m \cdot T_{1}-t_{1}-\sum^{K-1}_{n=1} (t_{n+1}-t_{n}) \\ & \leq u_{1}+(K-1)\cdot m\cdot T_{1}-t_{1}-(K-1) \cdot \min_{n\in \mathbb{N}} \{ t_{n+1}-t_{n}\} \\ & =u_{1}-t_{1}-(K-1)\cdot \Bigl(\min_{n\in \mathbb{N}} \{ t_{n+1}-t_{n}\}-m\cdot T_{1} \Bigr) \\ & \leq u_{1}-t_{1}-\max_{n\in \mathbb{N}} \{ \bigl[t_{n+1}-t_{n} \}-(c+T_{1}) \bigr] \\ & < t_{2}-t_{1}-\max_{n\in \mathbb{N}} \{ \bigl[t_{n+1}-t_{n}\}-(c+T_{1}) \bigr] \\ & = c+T_{1}+t_{2}-t_{1}-\max _{n\in \mathbb{N}} \bigl\{ [t_{n+1}-t_{n}] \bigr\} \\ &\leq c+T_{1}. \end{aligned}$$

 □

### Proof of Theorem 4.1

Part (a) is just part (a) of the previous lemma, while part (b) is a combination of parts (a), (c), and (d) of the lemma. For part (c) of the theorem, note that each $u_{n}$ is equal to some member of the sequence $\{s_{l}\}$ and by part (b), for any $n\geq K$, $u_{n}\in [t_{n}+c,t_{n}+c+T_{1})$. The result follows once we observe that there is a unique member of the sequence $\{s_{l}\}$ in each of these intervals. □

### Proof of Theorem 4.3

Note that $u_{n}$ is equal to the *n*th member of the sequence $s_{l}=(l-1)\cdot T_{1}+s_{1}=l\cdot T_{1}+(s_{1}-T_{1})$ that satisfies

36 $$ \overline{s_{l}}\geq c. $$

Thus, if we denote by $d_{n}$ the *n*th positive integer *l* for which Eq. (36) is satisfied, then $u_{n}=d_{n}\cdot T_{1}+(s_{1}-T_{1})$. Equation (36) can also be written as $\frac{s_{l}}{T_{2}}\mod 1\geq \frac{c}{T_{2}}$, or equivalently

37 $$ l\cdot \frac{T_{1}}{T_{2}}+\frac{s_{1}-T_{1}}{T_{2}}\mod 1\geq \frac{c}{T_{2}}. $$

Thus, by definition of $d_{n}$ we get

38 $$ n=\operatorname{card} \biggl\{ l\leq d_{n}:l\cdot \frac{T_{1}}{T_{2}}+ \frac{s_{1}-T_{1}}{T_{2}}\mod 1\geq \frac{c}{T_{2}} \biggr\} . $$

If $\frac{T_{1}}{T_{2}}\notin \mathbb{Q}$, by Weyl’s equidistribution theorem [26, pp. 105–113] we have

39 $$ \lim _{n\to \infty }\frac{n}{d_{n}}=1-\frac{c}{T_{2}}. $$

Therefore,

40 $$ \lim _{n\to \infty }\frac{n}{u_{n}}=\lim _{n\to \infty }\frac{d_{n}}{u_{n}}\cdot \lim _{n\to \infty }\frac{n}{d_{n}}= \frac{1}{T_{1}}\cdot \biggl( 1-\frac{c}{T_{2}} \biggr) . $$

If on the other hand $\frac{T_{1}}{T_{2}}=\frac{p}{q}$, where $p,q$ are relatively prime positive integers, then the left hand side of Eq. (37) is periodic with period *q*, taking *q* equally spaced values (at distances $\frac{1}{q}$ from each other). Let *z* be the number of these values that lie in the interval $[ \frac{c}{T_{2}},1 ) $. Then we have $\lim _{n\to \infty }\frac{n}{d_{n}}=\frac{z}{q}\approx 1-\frac{c}{T_{2}}$. More precisely, we have $( 1-\frac{c}{T_{2}} ) >\frac{z-1}{q}$ and $( 1-\frac{c}{T_{2}} ) <\frac{z+1}{q}$. From these two inequalities we get

41 $$ \biggl\vert \frac{z}{q}- \biggl( 1-\frac{c}{T_{2}} \biggr) \biggr\vert < \frac{1}{q}. $$

The proof is complete once we note that $\lim _{n\to \infty }\frac{n}{u_{n}}=\frac{1}{T_{1}}\cdot \lim _{n\to \infty }\frac{n}{d_{n}}= \frac{1}{T_{1}}\cdot \frac{z}{q}$. □

## Appendix B: Number-theoretic application of Theorem 4.1

Here we apply Theorem 4.1 to get a number-theoretic result. The importance of the result lies in the way it contrasts with Weyl’s equidistribution theorem, so we begin by stating the latter (for a proof see [26, pp. 105–113]).

### Theorem B.1

*Let* $\alpha \notin \mathbb{Q}$, $c\in [0,1]$, *and define*

42 $$ a_{n}=\textstyle\begin{cases} 1 & \textit{if } (n\cdot \alpha \mod 1)\geq c, \\ 0 & \textit{otherwise.}\end{cases} $$

*Then*

43 $$ \lim _{n\to \infty }\frac{\sum ^{n}_{i=1}a_{i}}{n}=1-c. $$

### Theorem B.2

*Let* $m\in \mathbb{N}$ *with* $m\geq 2$, $\alpha \in ( \frac{1}{m+1},\frac{1}{m} ) $, *and* $c\in [ 1-m\alpha,1-\alpha ) $, *and let the sequence* $a_{n}$ *be defined by*

44 $$ a_{n}= \textstyle\begin{cases} 1 & \textit{if } a_{n-1}=\cdots =a_{n-m+1}=0 \textit{ and } (n\cdot \alpha \mod 1)\geq c, \\ 0 & \textit{otherwise,}\end{cases} $$

*for* $n\geq 1$, *and* $a_{0}=a_{-1}=\cdots =a_{-m+2}=0$. *Then*

45 $$ \lim _{n\to \infty }\frac{\sum ^{n}_{i=1}a_{i}}{n}=\alpha. $$

### Proof

Let us consider the sequence $b_{n}=a_{n+k-1}$, where *k* is the smallest integer such that $k\cdot \alpha \geq c$, so that $b_{1}=1$. Note that since $1-c>\alpha $, there is at least one multiple of *α* in $[c,1)$, therefore, $k\cdot \alpha <1$. Let $s_{1}=k\cdot \alpha $. Then $s_{n}=(n+k-1)\cdot \alpha $. Note that $b_{n}=1$ if and only if $b_{n-1}=\cdots =b_{n-m+1}=0$ and $s_{n}\mod 1\geq c$.

We apply Theorem 4.1 with $T_{1}=\alpha $, $t_{n}=n-1$, and $m,c$ and $s_{1}$ as above. Note that the condition $s_{1}\in [c,t_{2})=[c,1)$ is satisfied, as shown above. The conclusion of Theorem 4.1 that we shall need is the fact that

46 $$ \lim _{n\to \infty }\frac{t_{n}}{u_{n}}=1, $$

where we recall that $u_{1}=s_{1}$ and

47 $$ u_{n}=\min_{l\in \mathbb{N}} \bigl\{ s_{l}:s_{l} \geq u_{n-1}+m \cdot T_{1} \text{ and } (s_{l} \mod 1)\geq c \bigr\} . $$

Let $d_{n}$ be the index of the *n*th non-zero term of the sequence $\{b_{l}\} $. We claim that $u_{n}=s_{d_{n}}$ for all $n\in \mathbb{N}$. We shall show this by induction on *n*. For the base case, note that $d_{1}=1$ and $u_{1}=s_{1}$. Suppose now that $u_{n}=s_{d_{n}}$ holds for some *n*. Then

48 $$\begin{aligned} u_{n+1} & =\min_{l\in \mathbb{N}} \{ s_{l}:s_{l}\geq s_{d_{n}}+m \cdot T_{1} \text{ and } s_{l} \mod 1\geq c\} \\ & =\min_{l\in \mathbb{N}} \{ s_{l}:s_{l}\geq s_{d_{n}+m} \text{ and } s_{l} \mod 1\geq c\} \\ & =\min_{l\in \mathbb{N}}\{ s_{l}:l\geq d_{n}+m \text{ and } s_{l} \mod 1\geq c\}. \end{aligned}$$

Therefore $u_{n+1}=s_{l'}$, where $l'$ is the smallest integer $l\geq d_{n}+m$ such that $s_{l}\mod 1\geq c$. By definition of $b_{n}$, $d_{n+1}$ is also the smallest integer $l\geq d_{n}+m$, such that $s_{l}\mod 1\geq c$. Therefore, $l'=d_{n+1}$ and this completes the inductive proof.

We may therefore substitute $u_{n}=s_{d_{n}}=(d_{n}+k-1)\cdot \alpha $, as well as $t_{n}=n-1$, in Eq. (46) to get $\lim _{n\to \infty }\frac{n-1}{(d_{n}+k-1)\cdot \alpha }=1$, thus $\lim _{n\to \infty }\frac{n}{d_{n}}=\alpha $. Therefore,

49 $$ \lim _{n\to \infty }\frac{\sum ^{n}_{i=1}a_{i}}{n}=\lim _{n\to \infty }\frac{\sum ^{n}_{i=1}b_{i}}{n}=\lim _{n\to \infty }\frac{\sum ^{d_{n}}_{i=1}b_{i}}{d_{n}}=\lim _{n\to \infty }\frac{n}{d_{n}}=\alpha, $$

where the second equality follows from the fact that $d_{n}$ is an unbounded increasing sequence and the third equality follows from the definition of $d_{n}$. □

## Appendix C: Modeling with a leaky integrate-and-fire neuron

In this section we show that the results of Sect. 3.4.3 can be reproduced with a leaky integrate-and-fire neuron in place of the Hodgkin–Huxley based SI cell.

Figure 25(a) shows a schematic of this cell with an inhibitory autapse and the two inputs, similar to the setting of Fig. 9(a). The cell is described by a single dynamic variable *V*, whose evolution is given by the equation

50 $$ \frac{dV}{dt}=-\frac{1}{\tau }(V-V_{\mathrm{rest}})+I, $$

with the extra rule that when *V* is greater than some value $V_{\mathrm{thresh}}$, we say that the neuron fires and *V* is reset to $V_{\mathrm{reset}}$.

The current *I* is a sum of two input currents, $I_{1}$ and $I_{2}$, and a synaptic current $I_{a}$. $I_{a}$ is set to −10 (mV/ms) when the cell fires and decays exponentially afterwards with time constant 40 ms. $I_{1}$ is a train of Dirac’s delta pulses of frequency $f_{1}=40\text{ Hz}$ and amplitude 35, with a random initial phase. $I_{2}$ is set to −10 at multiples of $T_{2}=\frac{1}{f_{2}}$, and decays exponentially thereafter with time constant 10 ms. We also set $V_{\mathrm{rest}}=-70$ mV, $V_{\mathrm{thresh}}=-40$ mV, $V_{\mathrm{reset}}=-100$ mV, and $\tau =10\text{ ms}$. These values are chosen so that the model satisfies conditions 1–3 and 7 of Sect. 3.5 and the interval of time that the cell does not fire after it has been inhibited is approximately the same as in the model of Fig. 9. As can be seen in Fig. 25(b) to Fig. 25(d), the behavior of this model is the same as the one in Fig. 9.

## Appendix D: Model parameters and simulations details

### D.1 Model parameters

Here we give the details of the parameters for the model described in Sects. 2.1 and 2.2 and for the various conditions. We begin with the functions appearing in Eqs. (1), (2) (Table 1—recall that the RS and IB cells are excitatory, while the FS and SI cells are inhibitory). We note that an equivalent way of describing the time constant $\tau _{x}$ and steady-state value $x_{\infty }$ of a first order differential equation is through the forward and backward rates $\alpha _{x}$ and $\beta _{x}$. The relation between the $( \tau _{x},x_{\infty } ) $ and the $( \alpha _{x},\beta _{x} ) $ descriptions is given by

51 $$\begin{aligned} \tau _{x}=\frac{1}{\alpha _{x}+\beta _{x}},\qquad x_{\infty }= \frac{\alpha _{x}}{\alpha _{x}+\beta _{x}},\qquad \alpha _{x}= \frac{x_{\infty }}{\tau _{x}},\qquad \beta _{x}= \frac{1-x_{\infty }}{\tau _{x}}. \end{aligned}$$

**Table 1:** Details for cell dynamics

Variable	$\tau _{x}$	$x_{\infty }$
(a)		
**m** (excitatory cell)	$0.25+4.35\cdot e^{-\frac{|\mathbf{V}+10|}{10}}$	$( 1+e^{\frac{-\mathbf{V}-29.5}{10}} ) ^{-1}$
**h** (excitatory cell)	$0.15+\frac{1.15}{1+e^{\frac{\mathbf{V}+33.5}{15}}}$	$( 1+e^{\frac{\mathbf{V}+59.4}{10.7}} ) ^{-1}$
**m** (inhibitory cell)	$0.25+4.35\cdot e^{-\frac{|\mathbf{V}+10|}{10}}$	$(1+e^{\frac{-\mathbf{V}-27}{11.5}} ) ^{-1}$
**h** (inhibitory cell)	$0.225+\frac{1.125}{1+e^{\frac{\mathbf{V}+37}{15}}}$	$(1+e^{\frac{\mathbf{V}+58.3}{6.7}} ) ^{-1}$
$\mathbf{m}_{AR}$	$(e^{-14.6-0.086\mathbf{V}}+e^{-1.87+0.07\mathbf{V}} )^{-1}$	$( 1+e^{\frac{\mathbf{V}-V_{0}}{5.5}} )^{-1}$

Variable	$\alpha _{x}$	$\beta _{x}$
(b)		
$\mathbf{m}_{KM}$	$\frac{0.02}{1+e^{\frac{-\mathbf{V}-20}{5}}}$	$0.01e^{\frac{-\mathbf{V}-43}{18}}$
$\mathbf{m}_{CaH}$	$\frac{4.8}{1+e^{-0.072(\mathbf{V}-5)}}$	$\frac{0.06(\mathbf{V}+8.9)}{e^{\frac{\mathbf{V}+8.9}{5}}-1}$

Function	Expression
(c)	
$m_{0}(\mathbf{V})$ (exc. cell)	$(1+e^{\frac{-\mathbf{V}-34.5}{10}} ) ^{-1}$
$m_{0}(\mathbf{V})$ (inh. cell)	$(1+e^{\frac{-\mathbf{V}-38}{10}} )^{-1}$

Expressions for functions involved in the dynamics of the membrane potential and the ionic current gating variables (Eqs. (1), (2)), taken from [12]. The expressions for $\tau _{KM}$, $\tau _{CaH}$, $m_{KM,\infty }$, and $m_{CaH,\infty }$ are given indirectly by the expressions in table (b) (see text). **V** is measured in mV. $V_{0}=-87.5$ for RS cells, and $V_{0}=-75$ for other types of cells/compartments.

As in [12], we make the following adjustments for particular cells/compartments, relative to the values in Table 1: for the IB axon, we multiply the forward rate $\alpha _{KM}$ of $\mathbf{m}_{KM}$ by 1.5 and its backward rate $\beta _{KM}$ by 1.25. For the RS cell, we multiply the forward rate of $\mathbf{m}_{AR}$ by 1.75 and the backward rate by 0.5. For the IB apical and basal dendrites, we multiply the forward rate of $\mathbf{m}_{AR}$ by 2.75, and its backward rate by 3, except for the simulations in Figs. 16(c), 17 and 19 to 21, where we multiply both the forward and backward rates by 3.

We give the parameter values for the various cells/compartments in Table 2, for the synapses in Table 3, for the electrical coupling in Table 4, for the inputs in Table 5, and the initial values used for the state variables in Table 6. We note that the time constants for the synapses that mediate the various inputs to the SI and FS cells are chosen to be equal to those of the synapses in the initial network that they are substituting. In particular, the input-1→FS and input-1→SI synapses have the same dynamics as the RS→FS and RS→SI synapses in the initial network, respectively. Similarly, the input-2→FS and input-2→SI synapses have the same dynamics as the IB→FS and IB→SI synapses, except in the simulations of Fig. 9, where the input-2→SI synapse mimics the FS→SI synapse.

**Table 2:** Cell parameters

var	RS	FS	SI	IB apical dendrite	IB basal dendrite	IB soma	IB axon
*J*	11	35	45	25.5	42.5	−4.5	−1.2
$g_{L}$	1	1	6	2	2	1	0.25
$g_{Na}$	200	200	200	125	125	50	100
$g_{K}$	20	20	10	10	10	10	5
$g_{AR}$	25	–	50	155	115	–	–
$g_{KM}$	–	–	–	0.75	0.75	–	1.5
$g_{CaH}$	–	–	–	–	–	6.5	6.5
$V_{L}$	−70	−65	−65	−70	−70	−70	−70
$V_{Na}$	50	50	50	50	50	50	50
$V_{K}$	−95	−100	−100	−95	−95	−95	−95
$V_{AR}$	−35	–	−35	−25	−25	–	–
$V_{KM}$	–	–	–	−95	−95	–	−95
$V_{CaH}$	–	–	–	125	125	–	–

Values for parameters appearing in Eq. (1), for the various cells/compartments. A dash means that the corresponding term is absent in that compartment. All values are taken from [12], except for the parameter *J* (all cells). For all cells/compartments: *C* = 0.9.

**Table 3:** Synapse parameters

synapse	*g*	$\tau _{r}$	$\tau _{d}$	$V_{0}$
RS→RS	0.5	0.25	1	0
RS→FS	3	0.25	1	0
RS→SI	5	2.5	1	0
FS→RS	125	0.5	5	−80
FS→FS	20	0.5	5	−75
FS→SI	8^∗^	0.5	6	−80
SI→RS^∗∗^	2.5	0.5	20	−80
SI→RS^∗∗∗^	0.7	0.25	1	0
SI→FS	4	0.5	20	−80
SI→SI	4	0.5	20	−80
SI→IB	4	0.5	20	−80
IB→FS	2	0.25	1	0
IB→SI	0.9	2.5	50	0
IB→IB	0.4	0.5	100	0

Values of parameters appearing in Eqs. (4) and (5) for the various synapses. The values for $\tau _{r}$, $\tau _{d}$, and $V_{rev}$ are taken from [12], whenever applicable. The synapse SI→FS does not appear in [12]; values for $\tau _{r}$, $\tau _{d}$, and $V_{rev}$ for this synapse are taken from [13]. All outgoing synapses from an IB cell are from the axon compartment. The target of the SI→IB synapse is the IB apical dendrite and that of the IB→IB synapse is the basal dendrite. ^∗^In Figs. 12(a) and 12(b), under the conditions with “weak FS→SI”, *g* = 5. ^∗∗^Networks of Figs. 1 and 3(a). ^∗∗∗^Network of Fig. 23(a).

**Table 4:** Electrical coupling parameters

electr. connection	g
$s\to d_{a}$	0.2
$s\to d_{b}$	0.2
*s*→*a*	0.3
$d_{a}\to s$	0.4
$d_{b}\to s$	0.4
*a*→*s*	0.3

Conductance of electrical connections of the various compartments of the IB cell. Although electrical coupling is two-way, each direction is considered separately in the model, having a source and a target. Each unidirectional electrical connection is included as a summand in the term $I_{el}$ of the target compartment. Conductance of connections in the opposite direction may differ, reflecting differences in the size of the two compartments. All values are taken from [12]. *s*: soma, $d_{a}$: apical dendrite, $d_{b}$: basal dendrite, *a*: axon.

**Table 5:** Input parameters

Figure/input	*σ*	Target	*g*	$\tau _{r}$	$\tau _{d}$
Figs. 3(b) and 3(c)	0.025	RS	150	0.2	0.5
Figs. 4, 14(a), 15 to 17 and 19 to 21	0.025^∗^	FS	2.5	0.25	1
		SI	5	2.5	1
Figs. 5 to 9, 12, 13 and 24, Input-1	0^∗∗^	FS	2.5	0.25	1
		SI	5^∗∗∗^	2.5	1
Figs. 5 to 8, 12 and 13, Input-2	0^∗∗^	FS	3	0.25	1
		SI	0.9	2.5	50
Figs. 9 and 24, Input-2	0	SI	8	0.5	6

Values of parameters appearing in Eqs. (4) and (6) for the various input-target combinations, as well as the parameter *σ* for the various inputs (see text after Eq. (7)). The frequency of input-1 and the frequency of the input in simulations with a unique input is 40 Hz, and the frequency of input-2 is 16.357 Hz, unless otherwise specified. In all cases $V_{0}=0$, except for input-2 in Fig. 9, where $V_{0}=-80$. ^∗^In Figs. 16, 19, 20 and 21, *σ* = 0.05, in Figs. 15 and 17*σ* = 0. ^∗∗^In Figs. 8, *σ* = 0.05 for both inputs. In Fig. 24, *σ* = 0.1 for input-1. ^∗∗∗^In Fig. 13, *g* = 10.

**Table 6:** Initial values for state variables

var	RS	FS	SI	IB ap. d.	IB bas. d.	IB soma	IB axon
**V**	−70/ − 60	−110/ − 100	−100/ − 90	−100/ − 90	−100/ − 90	−100/ − 90	−100/ − 90
**h**	0/0.05	0/0.05	0/0.05	0/0.05	0/0.05	0/0.05	0/0.05
**m**	0/0.05	0/0.05	0/0.05	0/0.05	0/0.05	0/0.05	0/0.05
$\mathbf{m}_{AR}$	0.035/0.06	–	0.02/0.06	0/0.001	0/0.001	–	–
$\mathbf{m}_{KM}$	–	–	–	0/0	0/0	–	0/0
$\mathbf{m}_{CaH}$	–	–	–	0/0.01	0/0.01	–	–

For each cell/compartment, the initial value is chosen randomly and independently from inside an interval. The values shown here are the minimum and maximum values of this interval.

The starting phases of the inputs were random, with the exception of input-2 in Fig. 24, where for the cell SI 1 it was started at 5 ms and for SI 2 and SI 3 after one third and two thirds of a period, respectively.

### D.2 Simulations and analysis of results

All simulations and analyses were performed in MATLAB [42].

Spike times were defined as the times that the membrane potential crossed the value 0 mV (similarly for timing of input pulses). For the IB axon, a spike was considered to initiate a new burst if the time from the previous spike was at least 7 ms, otherwise it was considered part of that burst.

The probability of a cell firing in response to an input-1 pulse, as a function of the time of arrival of this pulse with respect to the last input-2 pulse (Figs. 7(a), 8(c), 9(c), 10(b) and 25(c)) was calculated as follows: the “time from last input-2 pulse” was separated into bins of size 0.5 ms and the input-1 pulses that fell into each bin were identified. For each of these pulses, a cell (SI or FS) was considered to fire “in response to” the pulse, if it fired within the next 3 ms. The proportion of input-1 pulses in that bin in response to which each cell fired was calculated, and this number was defined as the probability of that cell firing in response to an input-1 pulse, for the corresponding input-2 phase (the center of the bin). The probability of a cell firing in response to an input-2 pulse, as a function of time of arrival with respect to last input-1 pulse (Figs. 7(b) and 8(d)) was calculated similarly.

All statistics were based on 20 sec simulations. To exclude any transients from the statistical analysis, the first 5 spikes/bursts (or input pulses) of the relevant cell were ignored, except in calculating average frequencies (Fig. 6 and similar graphs). The first five and last three spikes are also not shown in Fig. 19. In Figs. 15(b) and 17(a) the simulation time shown is between 1–5 sec.

## Footnotes

1 Extensions of the PRC theory, like second order PRC or functional PRC, were not applicable in our case. See Discussion.

2 Recall that mode-locking means that the frequencies are rationally related.

3 The fact that a later SI spike leads to a longer IB interburst interval is not surprising, since the SI cell is inhibitory. On the other hand, there are other currents that slowly build up (h-current) or diminish (M-current) during the interburst interval, and bring the IB cell closer to firing as time progresses. This results in a “regression to the mean” phenomenon, where a later SI spike delays the IB burst, but by a smaller amount than the delay in the SI spike itself.

4 $t_{0}$ itself is not of particular importance. We have chosen a specific time to measure the state in order to facilitate the presentation.

5 The dendrite M-currents are small in magnitude and might not affect significantly the firing of the cell.

6 We should also expect the response to depend on the form of the stimulus, but in our model (and very often in general) all action potentials of a cell have approximately the same form, thus also approximately the same effect on the synapses.
